# Microbial Diversity of Psychrotolerant Bacteria Isolated from Wild Flora of Andes Mountains and Patagonia of Chile towards the Selection of Plant Growth-Promoting Bacterial Consortia to Alleviate Cold Stress in Plants

**DOI:** 10.3390/microorganisms9030538

**Published:** 2021-03-05

**Authors:** Paulina Vega-Celedón, Guillermo Bravo, Alexis Velásquez, Fernanda P. Cid, Miryam Valenzuela, Ingrid Ramírez, Ingrid-Nicole Vasconez, Inaudis Álvarez, Milko A. Jorquera, Michael Seeger

**Affiliations:** 1 Molecular Microbiology and Environmental Biotechnology Laboratory, Department of Chemistry, Universidad Técnica Federico Santa María, Avenida España 1680, Valparaíso 2390123, Chile; bravoc.guillermo@gmail.com (G.B.); alexvelasquezsaez@gmail.com (A.V.); mvalenzuelao@yahoo.com (M.V.); ingrid94nicole@gmail.com (I.-N.V.); inaudisah@gmail.com (I.Á.); 2 Center of Biotechnology “Dr. Daniel Alkalay Lowitt”, Universidad Técnica Federico Santa María, General Bari 699, Valparaíso 2390136, Chile; ingrid.ramirez@usm.cl; 3 Laboratorio de Ecología Microbiana Aplicada (EMALAB), Departamento de Ciencias Químicas y Recursos Naturales, Universidad de La Frontera, Avenida Francisco Salazar 1145, Temuco 4811230, Chile; fernanda.cid.alda@gmail.com (F.P.C.); milko.jorquera@ufrontera.cl (M.A.J.); 4 Center of Plant-Soil Interaction and Natural Resources Biotechnology, Scientific and Technological Bioresource Nucleus (BIOREN), Universidad de La Frontera, Avenida Francisco Salazar 1145, Temuco 4811230, Chile

**Keywords:** psychrotolerant bacteria, plant growth-promoting bacteria, bacterial consortium, *Pseudomonas*, *Curtobacterium*, cold stress, tomato, Andes Mountain, Patagonia

## Abstract

Cold stress decreases the growth and productivity of agricultural crops. Psychrotolerant plant growth-promoting bacteria (PGPB) may protect and promote plant growth at low temperatures. The aims of this study were to isolate and characterize psychrotolerant PGPB from wild flora of Andes Mountains and Patagonia of Chile and to formulate PGPB consortia. Psychrotolerant strains were isolated from 11 wild plants (rhizosphere and phyllosphere) during winter of 2015. For the first time, bacteria associated with *Calycera*, *Orites*, and *Chusquea* plant genera were reported. More than 50% of the 130 isolates showed ≥33% bacterial cell survival at temperatures below zero. Seventy strains of *Pseudomonas*, *Curtobacterium*, *Janthinobacterium*, *Stenotrophomonas*, *Serratia*, *Brevundimonas*, *Xanthomonas*, *Frondihabitans*, *Arthrobacter*, *Pseudarthrobacter*, *Paenarthrobacter*, *Brachybacterium*, *Clavibacter*, *Sporosarcina*, *Bacillus*, *Solibacillus*, *Flavobacterium*, and *Pedobacter* genera were identified by 16S rRNA gene sequence analyses. Ten strains were selected based on psychrotolerance, auxin production, phosphate solubilization, presence of *nifH* (nitrogenase reductase) and *acdS* (1-aminocyclopropane-1-carboxylate (ACC) deaminase) genes, and anti-phytopathogenic activities. Two of the three bacterial consortia formulated promoted tomato plant growth under normal and cold stress conditions. The bacterial consortium composed of *Pseudomonas* sp. TmR5a & *Curtobacterium* sp. BmP22c that possesses ACC deaminase and ice recrystallization inhibition activities is a promising candidate for future cold stress studies.

## 1. Introduction

The production and quality of agricultural crops are decreased by a wide range of abiotic stresses, including cold and high temperatures, drought, and salinity. It is estimated that more than 50% of global yield loss for major agricultural crops is due to abiotic stress [1]. Every year agriculture worldwide is affected by low temperatures, which are more frequent in the last years due to climate change. In the United States of America, there are higher economic losses from frost damage than from any other weather-related phenomenon [2]. In Chile during the year 2013, frosts caused a reduction of 22% in exportable fruit, which represented a loss of more than US $800 million [3]. Climate change affects the natural systems, human health, and agricultural production [4], and with the increase in temperature, the cold acclimatization of plants occurs later in autumn or early winter [5]. This disorganized cold acclimatization causes a higher susceptibility of plants to erratic temperature events [5]. Due to climate change, extreme weather events, such as frost (<0 °C), are increasing. Therefore, cold stress is an important challenge for agriculture [3,6,7].

In this context, the plant growth-promoting microorganisms play an essential role for plants during their growth and adaptation to the changing environment through some mechanisms, such as nitrogen fixation, phosphate solubilization, modifying plant hormone levels, and improving plant defense responses to biotic and abiotic stresses [8,9,10,11]. The agricultural importance of cold-tolerant microorganisms arises because crops are subject to temporary cold periods, which are detrimental to mesophilic microbial processes associated with plant growth-promoting activities [12]. Plant growth-promoting bacteria (PGPB) can employ one or more different strategies to allow higher tolerance and additionally promote plant growth under abiotic stress [8]. The identification of PGPB that retain their potential to promote plant growth at low temperatures, is a worldwide trend in the field of agricultural inoculation technology [13,14]. On the other hand, the formulation of bacterial consortia (≥2 compatible bacteria) can increase the efficiency of agricultural crop production particularly under challenging environmental conditions, due to exploitation of their complementary and synergistic characteristics, compared to a single bacterium [15,16]. Various studies report plant growth-promoting effects with the application of a single bacterium, such as *Pseudomonas*, *Bacillus*, *Serratia*, *Pantoea*, and *Paraburkholderia* strains on canola, wheat, grapevine, and tomato under cold stress [17,18,19,20,21,22,23,24,25,26,27]. The formulation of bacterial consortia to alleviate cold stress in plants has been scarcely reported. A bacterial consortium formulated with *Bacillus* and *Brevibacillus* strains protects and promotes the growth of rice seedlings under cold stress [28]. Another consortium composed of *Bacillus* and *Serratia* strains promotes the survival of tomato plants subjected to cold stress [29].

The application of PGPB consortia is an eco-friendly alternative to agriculture that has the potential to increase crop yield under normal and stressful conditions. In addition, the use of PGPB reduces the application of chemical products, decreasing the environmental impact in agriculture (e.g., soil erosion), and increasing the microbial diversity and functionality of agricultural soils with the concomitant production of healthy and safe foods [16]. To search for novel bioproducts to alleviate the effects of cold stress towards a sustainable agriculture, Chile presents ideal extreme environments for the isolation of psychrotolerant bacteria associated with wild plants adapted to low-temperature conditions. Chile is a 4500 km long and narrow country that includes a wide variety of climates and extreme ecosystems, such as the Atacama Desert, Altiplano, the Andes Mountains, Patagonia, and Antarctica [30,31]. Psychrotolerant PGPB from areas with extreme environments such as Chilean Andes Mountains, Patagonia, and Antarctica, Canadian Arctic, and High Indian Himalayas have been isolated [17,19,20,21,22,26,30,32,33].

The aims of this study were to isolate and characterize psychrotolerant PGPB from the wild flora of Chile, and to formulate PGPB consortia. The bioprospecting of psychrotolerant PGPB with novel characteristics was carried out from wild plants that inhabit three areas of the Andes Mountains and one area of the Patagonia of Chile during the winter of 2015. These areas have low temperatures and frosts throughout the year [31,34]. The bacterial isolations were carried out at 4 °C, obtaining a total of 130 isolates. A scale of bacterial cell survival (BCS%) to temperatures below zero (−20 °C) was used to screening the psychrotolerant potential of the 130 strains. Seventy strains with high psychrotolerant potential were selected (≥33% BCS, high category). Bacterial isolates identified by 16S rRNA gene sequence analyses belong to 4 phyla (Proteobacteria, Actinobacteria, Firmicutes, and Bacteroidetes) and 18 different genera. Bacterial consortia were selected based on psychrotolerance, auxin production, phosphate solubilization, detection of *nifH* (nitrogenase reductase) and *acdS* (1-aminocyclopropane-1-carboxylate (ACC) deaminase) genes, and antimicrobial activities to phytopathogens. Three bacterial consortia were selected for tomato growth-promoting assays; two consortia promoted growth of tomato plants. Finally, the ACC deaminase and ice recrystallization inhibition activities allowed the selection of one bacterial consortium composed of *Pseudomonas* sp. TmR5a & *Curtobacterium* sp. BmP22c (BC3), which possess diverse plant growth-promoting activities. The BC3 is an attractive candidate for future plant frost protection assays.

## 2. Materials and Methods

### 2.1. Chemicals, Reagents, and Culture Media

Indole-3-acetic acid (IAA; 98% purity), *L*-tryptophan (>99% purity), α-ketobutyrate (97% purity), and 2,4-dinitrophenylhydrazine (97% purity) were purchased from Sigma-Aldrich (Darmstadt, Germany). Cycloheximide was obtained from United States Biological (Salem, MA, USA). Sulfuric acid, toluene, sodium chloride, sodium hydroxide, glucose, sucrose, glycerol, gluconic acid, citric acid, KH_2_PO_4_, Na_2_HPO_4_, MgSO_4_ × 7H_2_O, FeSO_4_ × 7 H_2_O, H_3_BO_3_, MnSO_4_ × H_2_O, ZnSO_4_ × 7H_2_O, CuSO_4_ × 5H_2_O, MoO_3_, FeCl_3_ × 6H_2_O, and (NH_4_)_2_SO_4_ were obtained from Merck (Darmstadt, Germany). Yeast extract, Tryptone, Bacto Proteose Peptone No. 3, Tryptic Soy Broth (TSB), Tryptic Soy Agar (TSA), and Müller-Hinton (MH) were purchased from Difco Laboratories (Franklin Lakes, NJ, USA); 1-aminocyclopropane-1-carboxylate (ACC) was obtained from Calbiochem (San Diego, CA, USA). Type III AFP was purchased from A/F Protein, Inc. (Waltham, MA, USA). GoTaq Green Master Mix was purchased from Promega (Madison, WI, USA). Bacterial Protein Extraction Reagent (B-PER) and Halt protease inhibitor cocktail were obtained from Thermo Fisher Scientific (Waltham, MA, USA). Quick Start Bradford Dye was purchased from Bio-Rad Laboratories (Irvine, CA, USA). Primers (Table 1) were obtained from Integrated DNA Technologies (Coralville, IA, USA).

### 2.2. Model and Phytopathogenic Bacterial Strains

The following model strains were used in this study: *Paraburkholderia xenovorans* LB400 (positive control for IAA biosynthesis) [38], *Pseudomonas protegens* CHA0 (plant growth-promoting bacterium with biocontrol activity) [39,40], *Escherichia coli* JM109 (negative control for ice recrystallization inhibition activity) [41], and *Achromobacter* sp. 188 (positive control for ACC deaminase activity) [42]. The phytopathogenic strains used in this study are *Pseudomonas syringae* pv. *syringae* Cc1 (*Pss*, ice nucleation active (INA) bacterial strain and a causal agent of bacterial canker in cherry trees), *Pectobacterium carotovorum* NCPPB 312 (*Pc*, formerly *Erwinia carotovora*, INA bacterial strain and pathogen in several crops) [43], *Clavibacter michiganensis* subsp. *michiganensis* OP3 (*Cmm*, tomato pathogen) [44], and *Agrobacterium tumefaciens* C58C1 (*At*, causal agent of crown galls on a wide range of plants) [43]. These strains were obtained from the culture collections of the Molecular Microbiology and Environmental Biotechnology Laboratory and Center of Biotechnology “Dr. Daniel Alkalay Lowitt” (Universidad Técnica Federico Santa María, Valparaíso, Chile) and the Applied Microbial Ecology Laboratory (Universidad de La Frontera, Temuco, Chile).

### 2.3. Collection of Wild Flora Samples

**Figure 1:**
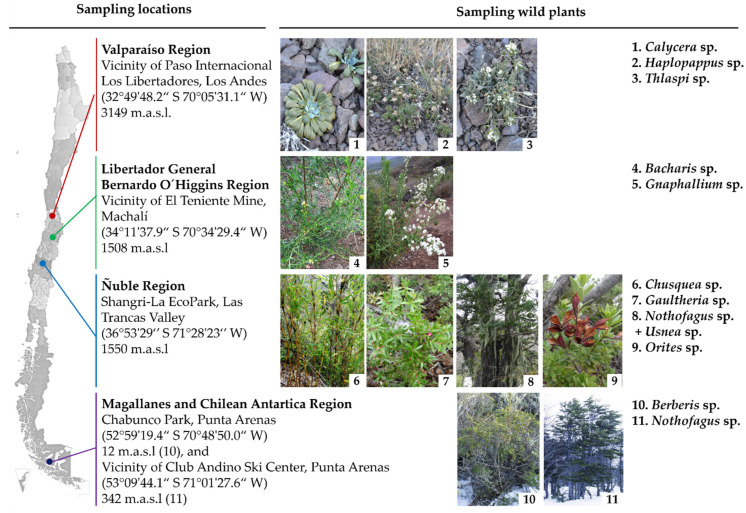
Location of sampling sites and sampled wild flora of Andean Mountains and Patagonia in Chile.

The wild flora samplings were carried out in four regions of Chile during the winter of 2015 (Figure 1). From the area of Paso Internacional Los Libertadores (32°49′48.2″ S 70°05′31.1″ W), Los Andes, Valparaíso Region at 3149 m above soil level (m.a.s.l.) three plants were sampled: *Haplopappus* sp., *Thlaspi* sp., and *Calycera* sp. From the vicinity of El Teniente Mine (34°11′37.9″ S 70°34′29.4″ W), Machalí, Libertador General Bernardo (L.G.B.) O’Higgins Region at 1508 m.a.s.l, two plants were sampled: *Baccharis* sp. and *Gnaphalium* sp. From Shangri-La EcoPark (36°53′29″ S 71°28′23″ W), Las Trancas Valley, Ñuble Region at 1550 m.a.s.l, four plants were sampled: *Orites* sp., *Gaultheria* sp., *Chusquea* sp., and *Nothofagus* sp. in association with the lichen *Usnea* sp. From Chabunco Park (52°59′19.4″ S 70°48′50.0″ W), Punta Arenas, Magallanes, and Chilean Antarctica (C.A.) The region at 12 m.a.s.l, one plant was sampled: *Berberis* sp. and from the vicinity of Club Andino Ski Center (53°09′44.1″ S 71°01′27.6″ W) Punta Arenas, Magallanes and C.A. Region at 342 m.a.s.l., one plant was sampled: *Nothofagus* sp. samples (only phyllosphere). A sampling of rhizosphere (near the root) and phyllosphere (leaves, stems, flowers, fruits) were performed in triplicate (*n* = 3) for each plant. The samples were kept in a close sterile plastic bag on ice and transported to the laboratory, where the samples were stored at 4 °C until analysis.

### 2.4. Psychrotolerant Bacteria Isolation

For bacterial isolation, the protocols from Kucheryava et al. [45] and Barrientos-Díaz et al. [46] with modifications were used. Four grams of soil near to the root or 4 g of a mixture of leaves, stems, flowers, or fruits of each sampled plant were mixed with 40 mL of saline solution (NaCl 0.85% *w v*^−1^) and stirred for 1 h in a Multi Reax vibrating shaker (Heidolph Instruments, Schwabach, Germany). Then, the suspensions were serially diluted (10^−1^, 10^−2^, 10^−3^, and 10^−4^) and 100 µL of each dilution were spread on TSA (pH 7) plates, supplemented with cycloheximide (100 µg mL^−1^) to inhibit the growth of fungi. The plates were incubated at 4 °C for 2 weeks. Morphologically distinct individual colonies were individually seeded on fresh TSA plates at least twice to isolate pure strains. The colonies of pure cultures were grown in TSA medium at room temperature (22 ± 3 °C) for 24–48 h, and stored in 15% glycerol (*v v*^−1^) at −20 and −80 °C.

### 2.5. Psychrotolerant Potential Test

The psychrotolerant potential was evaluated with a bacterial cell survival test at subzero temperatures described by Sun et al. [17], with modifications. The isolates were cultured in 200 µL TSB in a 96-well microplate for 72 h at 20 °C and then subjected to −20 °C for 24 h. Colony-forming units (CFU) per mL were counted using the microdroplet method [47], before and after the cell freezing treatment. The bacterial cell survival percentage (BCS%) was calculated. All assays were performed with 3 independent replicates. Five categories of BCS% were assigned: low (<10%), moderate (10–32%), high (33–55%), very high (56–78%), and excellent (79–100%). Psychrotolerant bacteria selection were carried from ≥33% BCS (high), based on the value obtained by the model PGPB *Pseudomonas protegens* CHA0 (30.7% BCS).

### 2.6. Genomic DNA Extraction, Identification of Selected Bacteria, and Phylogenetic Analysis

For the identification of the 70 selected psychrotolerant bacteria (≥33% BCS), the 16S rRNA gene was amplified, using the universal primers 27F and 1492R (Table 1). The extraction of genomic DNA from selected bacteria was carried out according to the protocol of Méndez et al. [48], with modifications. A growth loop of each bacterium was resuspended in 100 μL of sterile Milli-Q water, incubated at 95 °C for 15 min and then at −20 °C for 15 min, centrifuged briefly and the supernatant was transferred to a clean tube and stored at −20 °C for further analysis. The reaction mix (50 µL) contained 1 µL of genomic DNA, 25 µL of GoTaq Green Master Mix, and 0.2 µM of each primer. The PCR reactions were carried out in an Eppendorf Mastercycler gradient thermal cycler (Hamburg, Germany). The reaction started with an initial denaturation of DNA at 95 °C for 5 min, followed by 30 cycles of denaturation at 95 °C for 1 min, alignment at 55 °C for 1 min, and elongation at 72 °C for 1.5 min, with a final extension at 72 °C for 10 min. The PCR products were sent to Macrogen Inc. (Seoul, Korea) for purification and sequencing using the 800R conserved universal primer. The sequences obtained were edited manually with Vector NTI Advance 11.5.5 and nucleotide analyses were performed by BLASTn in the National Center for Biotechnology Information (NCBI) server. From the 16S rRNA gene sequence analyses, the bacteria were identified at the genus level. The phylogenetic analysis was performed to study the evolutionary relationships of the sequences based on the alignments calculated by CLUSTAL W using the default options. The evolutionary history was inferred using the Neighbor-Joining method [49]. Evolutionary analyses were conducted in MEGA 5.2.2 software [50]. In addition, the partial 16S rRNA gene sequences of the bacteria were deposited in GenBank under the accession numbers MW548335-MW548404.

### 2.7. Bacterial Consortia Selection

For the preliminary selection of bacterial consortia, the following activities were determined. First, (i) auxin production; followed by (ii) phosphate solubilization, and detection of *nifH* and *acdS* genes, and finally (iii) antimicrobial activity against phytopathogenic bacteria.

#### 2.7.1. Auxin Production

Auxin production was determined by Salkowski colorimetric method [51,52] for the 70 selected strains. Bacterial cultures (TSB medium ~12 h, turbidity at 600 nm of 1) were incubated at 20 °C in TSB medium supplemented with *L*-tryptophan (500 µg mL^−1^) for 48 h [53]. Subsequently, the cultures were centrifuged at 21,000× *g* in a Rotina 380R centrifuge (Hettich, Westphalia, Germany). The supernatant (0.5 mL) was mixed with 2 mL of Salkowski’s reagent (150 mL of concentrated H_2_SO_4_, 250 mL of distilled H_2_O, 7.5 mL of 0.5 M FeCl_3_ × 6H_2_O) and was incubated for 20 min at room temperature before measuring the absorbance at 535 nm, in a Jenway 6320D spectrophotometer (Stone, England). The auxin concentration in each sample was calculated based on a standard curve that ranged from 1 to 20 µg mL^−1^ of IAA. *Paraburkholderia xenovorans* LB400 was used as a positive control for IAA production [38]. This test was repeated three times for each bacterial strain. Bacteria were selected under the following criteria: [Auxin] (µg mL^−1^)/Turbidity_600nm_ ≥ 0.5 µg mL^−1^ and/or BCS ≥50%.

#### 2.7.2. Phosphate Solubilization and Detection of the *nifH* and the *acdS* Genes

For the 41 selected bacterial strains, the phosphate solubilization was analyzed by visualizing solubilization halos in Pikovskaya agar medium [54]. Bacterial cultures in TSB medium ~12 h, were adjusted to a turbidity at 600 nm of 1, and 10 µL of each bacterial isolate were plated in quadruplicate on plates with Pikovskaya agar medium. The plates were incubated at room temperature for 1 week. Bacterial isolates with a positive solubilization result were selected for evaluation of phosphate solubilization at 4 °C. *Pseudomonas protegens* CHA0 was used as a positive control for phosphate solubilization [40]. The results of phosphate solubilization were evaluated by photographic records. Positive bacteria showed a solubilization halo around the macrocolony.

The amplification of *nifH* (nitrogenase reductase) and *acdS* (ACC deaminase) genes were carried out using the primers nifH-F-Rösch and nifH-R-Rösch [36], and DegACC-F and DegACC-R [37], respectively (Table 1). The reaction mix (12.5 µL) contained 1 µL of genomic DNA, 6.25 µL of GoTaq Green Master Mix, and 0.2 µM of each primer. The PCR reactions were carried out in an Eppendorf Mastercycler gradient thermal cycler (Hamburg, Germany). The reaction for *nifH* gene detection was performed with an initial denaturation of DNA at 95 °C for 5 min, followed by 35 cycles of denaturation at 95 °C for 30 s, alignment at 55 °C for 30 s, and elongation at 72 °C for 30 s, with a final extension at 72 °C for 5 min. The reaction for *acdS* gene detection started with an initial denaturation of DNA at 95 °C for 5 min, followed by 35 cycles of denaturation at 95 °C for 1 min, alignment at 50 °C for 1 min, and elongation at 72 °C for 40 s, with a final extension at 72 °C for 7 min. For detection of both genes, amplification of the 16S rRNA gene was additionally carried out as a positive control. The genomic DNA of *P. xenovorans* LB400 was used as a positive control for the detection of both genes.

Twenty-one bacterial isolates that showed multiple activities/determinations were selected.

#### 2.7.3. Antimicrobial Activities against Phytopathogenic Bacteria

Antimicrobial activities of the 21 selected bacterial strains were carried out by the radial streak method according to Coman et al. [55], with modifications. The antimicrobial activity against the phytopathogenic bacteria *Pseudomonas syringae* pv. *syringae* Cc1, *Pectobacterium carotovorum* NCPPB 312, *Clavibacter michiganensis* subsp. *michiganensis* OP3, and *Agrobacterium tumefaciens* C58C1 was evaluated twice per duplicate. Selected bacteria were grown on Yeast Malt (YM; 10 g L^−1^ glucose, 3 g L^−1^ malt extract, 5 g L^−1^ peptone, 3 g L^−1^ yeast extract) and MH (Difco Laboratories) media for ~12 h at 25 °C. Then the inoculum was adjusted to turbidity at 600 nm of 1, and 100 µL of each isolate were deposited in the center of the plate, air drying and incubated for 72 h at room temperature. The phytopathogenic bacteria were previously grown in YM and MH media for ~12 h and were adjusted to turbidity at 600 nm of 1. Then, 10 µL of each phytopathogenic bacteria were arranged in radial lines surrounding growth of selected bacteria and incubated at room temperature. *Pseudomonas protegens* CHA0 was used as a positive control for antimicrobial activity.

Ten bacterial isolates that exhibited the highest microbial growth inhibition were selected for the formation of the bacterial consortia.

#### 2.7.4. Compatibility Tests and Formulation of Bacterial Consortia

The compatibility tests through a direct method among the 10 selected bacterial strains were carried out twice per duplicate. The selected bacteria were grown in YM and MH media for ~12 h at 25 °C. Then the inocula of the strains were adjusted to turbidity at 600 nm of 0.3 and 0.6. A volume of 100 µL (0.3) were spread throughout the plate and 10 µL (0.6) drops were placed in plates of each strains grown previously and incubated at room temperature. Inhibitions were observed at 24, 48, and 72 h.

Three bacterial consortia formulated with two bacterial strains (BC1, BC2, and BC3) were selected according to their compatibility (absence of inhibition halo) and in accordance with those that presented the highest values of all the activities/determinations.

### 2.8. Plant Growth-Promoting Assays with Bacterial Consortia

#### 2.8.1. Effect of Bacterial Consortia on Tomato Plants Growth

To determine the plant growth-promoting effect of the three selected bacterial consortia, a pot assay with tomato plants (*Solanum lycopersicum* L. cv. San Pedro) was evaluated. Tomato seeds were germinated for 2 weeks in a pot with peat as substrate. Then, the tomato seedlings were placed individually in pots (0.52 L) with 100 g peat and treated with bacterial consortia every 2 weeks for 2 months in a growth chamber at 25 °C with a photoperiod of 16 h light and 8 h dark, with seven replicates each treatment. The bacterial isolates of each bacterial consortia (BC1, BC2, and BC3) were mixed in the same concentration ratio in distilled water, obtaining a final bacterial concentration of 2 × 10^8^ CFU mL^−1^. The consortia were applied in a foliar and radicular treatment, soaking all the plant with a concentration of 2 × 10^8^ CFU mL^−1^ [56] and inoculated on the base of the plant with a final concentration on soil of 2 × 10^6^ CFU g^−1^ [57] in each application. The same procedure was carried out for the negative (NC) and positive (PC) controls, using only distilled water and the commercial biostimulant Nutrisac (Anasac, Santiago, Chile) based on amino acids and nitrogen, respectively. Nutrisac was used and diluted according to the manufacturer’s instructions. At the end of the trial, the number of leaves, stem diameter, plant height, and fresh weight of the aerial part were measured.

#### 2.8.2. Effect of Bacterial Consortia on Tomato Seeds Germination

To determine the plant growth-promoting effect of the three selected bacterial consortia, an in vitro germination test of tomato seeds (*Solanum lycopersicum* L. cv. San Pedro) was evaluated at 14 and 25 °C. A biopriming assay was performed as described by Subramanian et al. [25], with modifications. For disinfection, tomato seeds were submerged in 70% ethanol for 30 s, then with sodium hypochlorite solution (1%) for 10 min, and subsequently washed three times with sterile distilled water. The bacteria of each consortia were mixed in the same concentration ratio in sterile distilled water, obtaining a final bacterial concentration of 2 × 10^8^ CFU mL^−1^. Sterile distilled water was used as a negative control. The positive control (Nutrisac) was used and diluted according to the manufacturer’s instructions. The disinfected tomato seeds were immersed in 10 mL of the different treatments (BC1, BC2, BC3, PC, and NC) for 4 h at 20 °C with shaking at 200 rpm, and then 10 seeds were placed in sterile plates with filter paper soaked with 1 mL of sterile distilled water (3 replicates for each treatment). This test was carried out at two different temperatures, 14 and 25 °C for 12 days in the dark. At the end of the test, the germination percentage and root length were measured in tomato seeds at 14 °C, and complete germination % (root + hypocotyl), root length, and hypocotyl length were evaluated in tomato seeds at 25 °C.

Two bacterial consortia that showed plant growth-promoting effects were selected.

### 2.9. Bacterial Consortium Selection

For the selection of one bacterial consortium, ACC deaminase and ice recrystallization inhibition (IRI) activities were determined.

#### 2.9.1. ACC Deaminase Activity

The ACC deaminase enzyme converts ACC into α-ketobutyrate (α-KB) and ammonium. The bacterial strains of selected consortia were analyzed to determine their ability to grow in culture medium with ACC as the sole nitrogen (N) source, according to the procedures described by Penrose and Glick [58] and Barra et al. [42]. Selected bacteria were grown in TSB medium for ~12 h at 30 °C. The bacterial cells were washed two times with saline solution (NaCl 0.85% *w v*^−1^) and centrifuged at 8000× *g* for 10 min at 4 °C. A volume of 500 µL of the culture was resuspended in saline solution and incorporated in 4.5 mL of DF (Dworkin and Foster) medium (4 g L^−1^ KH_2_PO_4_, 6 g L^−1^ Na_2_HPO_4_, 0.2 g L^−1^ MgSO_4_ × 7H_2_O, 2 g L^−1^ gluconic acid, 2 g L^−1^ citric acid, and 1 mL trace elements containing 0.001 g L^−1^ FeSO_4_ × 7H_2_O, 0.01 g L^−1^ H_3_BO_3_, 0.011 g L^−1^ MnSO_4_ × H_2_O, 0.125 g L^−1^ ZnSO_4_ × 7H_2_O, 0.078 g L^−1^ CuSO_4_ × 5H_2_O, 0.01 g L^−1^ MoO_3_; with (NH_4_)_2_SO_4_ (2 g L^−1^) as sole N source and glucose (2 g L^−1^) as the carbon source) for 1–2 days at 30 °C. The bacterial cells were washed three times with saline solution and centrifuged at 8000× *g* for 10 min at 4 °C. The bacterial pellets were resuspended in 1 mL of saline solution and 100 µL of each inoculum were incorporated in 3 mL of DF medium with 3 mM ACC as sole N source and glucose as carbon source, in triplicate. Growth was evaluated for 5 days at 30 °C.

The ACC deaminase activity was determined by measuring µmoles α-KB mg protein^−1^ h^−1^ produced in the assays. The procedure described was carried out again for bacteria with positive growth in ACC, to evaluate ACC deaminase activity. A volume of 250 µL of inoculum was incorporated into 7.5 mL of DF medium with 3 mM ACC as sole nitrogen source and glucose as carbon source. On the fourth day, the cells were washed two times with saline solution and centrifuged at 8000× *g* for 10 min at 4 °C. Bacteria pellets were resuspended in 1 mL of 0.1 M Tris-HCl pH 7.6 and centrifuged at 16,000× *g* for 5 min, the supernatant was removed, and the pellet was resuspended in 600 µL of 0.1 M Tris-HCl pH 8.5. A volume of 30 µL of toluene was added and stirred for 30 s, and a volume of 100 µL was used to measure the protein concentration by Bradford method [41]. A volume of 200 µL (in duplicate) was placed in a new centrifuge tube, 20 µL of 0.5 M ACC was added, shaken briefly, and incubated for 15 min at 30 °C. A volume of 1 mL of 0.56 M HCl was added, it was stirred and centrifuged for 5 min at 16,000× *g* at room temperature. A volume of 1 mL of supernatant was taken, 800 µL of 0.56 M HCl was added, stirred, and placed in glass tubes to later incorporate 300 µL of 2,4-dinitrophenylhydrazine (0.2% 2,4-dinitrophenylhydrazine in 2 M HCl), stirred and incubated at 30 °C for 30 min. A volume of 2 mL of 2 N NaOH was added and it was stirred. Then, the absorbance at 540 nm was measured on a Multiskan GO spectrophotometer (Thermo Fisher Scientific, Waltham, MA, USA). A standard curve of ɑ-KB from 0 to 2 mM was performed. The µmoles α-KB mg protein^−1^ h^−1^ were calculated according to the values of α-KB and the protein concentration (mg mL^−1^) after an incubation of 15 min.

#### 2.9.2. IRI Activity

For bacterial strains of selected consortia, IRI activity was evaluated according to the protocol of Cid et al. [41] and Gilbert et al. [59] with modifications. The bacteria were grown in 5 mL of TSB at 4 °C for 10 days without agitation. Bacterial cells were centrifuged at 5000× *g* for 10 min at 4 °C and gently resuspended (up and down, to avoid generating bubbles) with 200 µL of B-PER in 2 mL Eppendorf tubes for protein extraction. A volume of 200 µL of sterile distilled water was added and gently homogenized. It was centrifuged at 24,000× *g* for 20 min at 4 °C. The supernatant was transferred to a fresh Eppendorf tube and 4 µL of a Halt protease inhibitor cocktail (10 µL mL^−1^) was added. A volume of 100 µL was used to measure the protein concentration by Bradford method [41]. Protein concentrations were adjusted to 1 mg mL^−1^ with a solution of B-PER: sterile distilled water (1:1). A volume of 50 µL of protein from each bacterium was mixed with 50 µL of a sucrose solution (60% *w v*^−1^) and this volume (100 µL) was placed in a 96-well plate. This step was performed six times for each protein extract of each strain. The plate was placed at −50 °C for 20 min, removed, and observed that all wells were frozen, otherwise, a small blow was provided to accelerate freezing. The plate was then transferred to −6 °C for 48 h, and at 24 h it was verified that the wells were still frozen. *Escherichia coli* JM109 was used as a negative control for IRI activity. The strain JM109 was grown for 1 week at room temperature plus 3 days at 4 °C. The antifreeze protein AFP type III was used as a positive control, and 30% *w v*^−1^ sucrose solution was used as blank. After 48 h, the absorbance at 500 nm was measured in a Multiskan GO spectrophotometer. The bacterial strains that presented a significantly higher absorbance than that obtained with the strain JM109 protein extract were considered IRI positive as reported by Cid et al. [41].

### 2.10. Statistical Analysis

One-way ANOVA were used to analyze the main effects. After carrying out one-way ANOVA, the Fisher’s LSD test was used to detect significant differences (*p* < 0.05) among the treatments. Data were analyzed using the non-parametric Kruskal–Wallis test if transformational data did not satisfy the assumption of homogeneity of variance. The Kruskal–Wallis test was followed by all pairwise multiple comparisons.

## 3. Results

### 3.1. Collection of Wild Flora from Andean and Patagonia Areas of Chile and Isolation of Psychrotolerant Bacteria

One hundred thirty isolates were obtained from 11 plants sampled in four regions belonging to the Andean and Patagonian zones of Chile (Figure 1). One hundred three strains were isolated from the rhizosphere, whereas twenty-seven strains belong to the phyllosphere of plants (Table 2).

### 3.2. Selection of Psychrotolerant Bacteria

The psychrotolerance of the 130 isolates was evaluated. Thirteen strains showed low, 47 moderate, 18 high, 24 very high, and 28 excellent psychrotolerance (Table 2). Seventy isolates with higher psychrotolerance (≥33% BCS) were selected for identification by 16S rRNA gene sequencing.

Of the 70 selected isolates, 49 belong to the rhizosphere (70%) and 21 were isolated from the phyllosphere (30%) (Table 2). Thirty-nine strains were isolated in Valparaíso Region, 14 bacterial strains in Liberator General Bernardo O’Higgins Region, eight strains in Ñuble Region, and nine strains in Magallanes and Chilean Antarctica Region. Valparaíso Region presented the highest average number of psychrotolerant isolates per plant, followed by Magallanes and Chilean Antarctica Region, Libertador General Bernardo O’Higgins Region, and Ñuble Region.

### 3.3. Identification of Selected Psychrotolerant Bacteria

**Figure 2:**
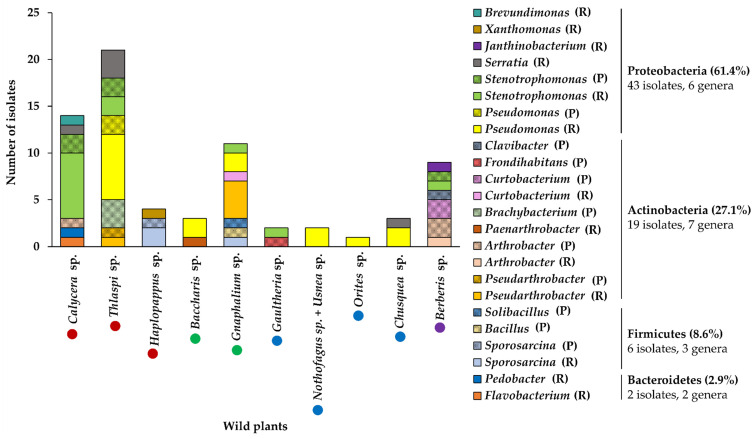
Biodiversity of the selected psychrotolerant bacterial strains from wild plants of Andean and Patagonia areas of Chile. The different genera were represented in solid colors for isolates from rhizosphere (R) and crosshatch colors for isolates from the phyllosphere (P). Circles next to the wild plant genera represent the regions of Chile. Red circles, Valparaíso Region; green circles, Libertador General Bernardo O’Higgins Region; blue circles, Ñuble Region; and purple circle, Magallanes and Chilean Antarctica Region.

The 70 selected isolates were identified through the analysis of the partial 16S rRNA gene sequence (Figure 2). The isolates belong to four bacterial phyla. The bacterial phylum with the highest diversity of genera was Actinobacteria with 19 identified strains from seven genera (*Pseudarthrobacter*, *Arthrobacter*, *Brachybacterium*, *Curtobacterium*, *Paenarthrobacter*, *Frondihabitans*, and *Clavibacter*), followed by Proteobacteria phylum with 43 identified strains from six genera (*Pseudomonas*, *Stenotrophomonas*, *Serratia*, *Janthinobacterium*, *Xanthomonas*, and *Brevundimonas*), Firmicutes phylum with six identified strains from three genera (*Sporosarcina*, *Solibacillus*, and *Bacillus*), and Bacteroidetes phylum, with two identified strains from two genera (*Flavobacterium* and *Pedobacter*). The most abundant isolates corresponded to *Pseudomonas* (18), *Stenotrophomonas* (17), *Pseudarthrobacter* (6), *Serratia* (5), *Arthrobacter* (4), and *Sporosarcina* (4) genera. Strains from *Pseudomonas*, *Stenotrophomonas*, *Sporosarcina*, *Curtobacterium*, *Arthrobacter*, and *Pseudarthrobacter* genera were isolated from rhizosphere and phyllosphere. On the other hand, *Serratia*, *Xanthomonas*, *Paenarthrobacter*, and *Janthinobacterium* genera were only isolated from rhizosphere, while *Solibacillus*, *Bacillus*, *Brachybacterium*, *Frondihabitans*, and *Clavibacter* genera were only isolated from the phyllosphere.

Of the 70 isolates, only two were closely related to phytopathogenic bacteria. The strain HsR18 that was isolated from the rhizosphere of *Haplopappus* sp. is closely related to *Xanthomonas translucens* [60,61]. The strain BmP21 that was isolated from the phyllosphere of *Berberis* sp. is closely related to *Clavibacter michiganensis* [44,62,63]. Both strains HsR18 and BmP21 were discarded for further analysis.

### 3.4. Auxin Biosynthesis of Selected Strains

**Figure 3:**
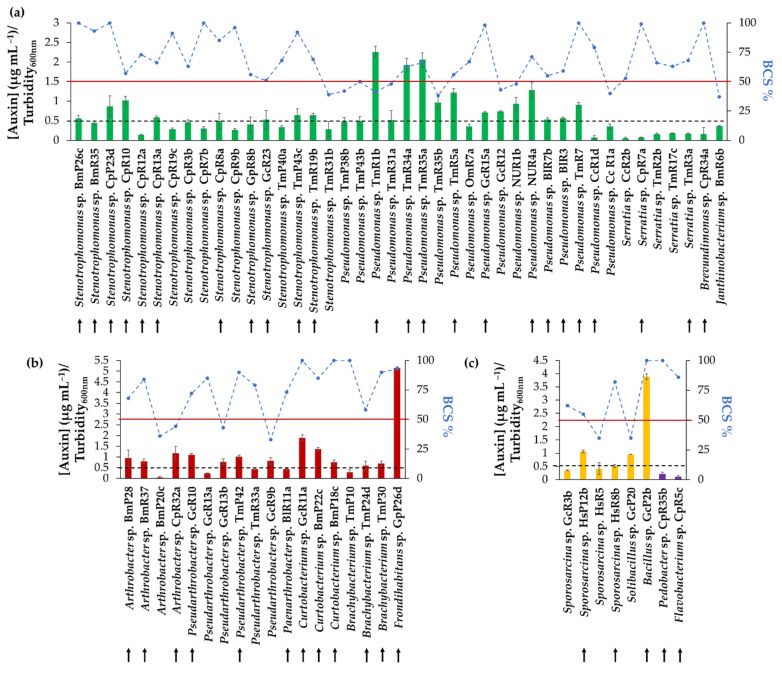
Auxin production and bacterial cell survival percentage (BCS%) of bacterial strains. (**a**) Auxin production and BCS% of Proteobacteria (green bars). (**b**) Auxin production and BCS% of Actinobacteria (red bars). (**c**) Auxin production and BCS% of Firmicutes (yellow bars) and Bacteroidetes (purple bars). The blue dots (joined by dotted lines) represent BCS% of each isolate. The solid red line is the 50% BCS value. The dashed black line represents the value of [Auxin] (µg mL^−1^)/Turbidity_600nm_ = 0.5 µg mL^−1^. Small black arrows indicate selected strains. Each value is a mean ± SD of three independent trials.

The capability to synthesize auxins in the presence of the precursor tryptophan was evaluated in the 68 selected strains (Figure 3). It was determined that 38 strains synthesize auxins, using the criterion of [Auxins]/Turbidity_600nm_ ≥0.5. Forty-one strains were selected based on auxin synthesis or BCS ≥50%. Strains of the same genus with similar activities and origins were removed for the following assays.

### 3.5. Phosphate Solubilization and Presence of the nifH and the acdS Genes in Selected Strains

The phosphate solubilization activity and the presence of the *nifH* and the *acdS* genes were analyzed in 41 selected strains. Fifteen strains showed phosphate solubilization activity at room temperature and 12 of these strains showed phosphate solubilization activity at 4 °C (Table 3). On the other hand, 35 and 21 strains were amplified for the *nifH* and the *acdS* genes, respectively (Table 3). Twenty-one of the 41 strains were selected based on the presence of a higher number of positive solubilization activity or PCR reactions.

### 3.6. Antimicrobial Activities of Selected Strains

**Figure 4:**
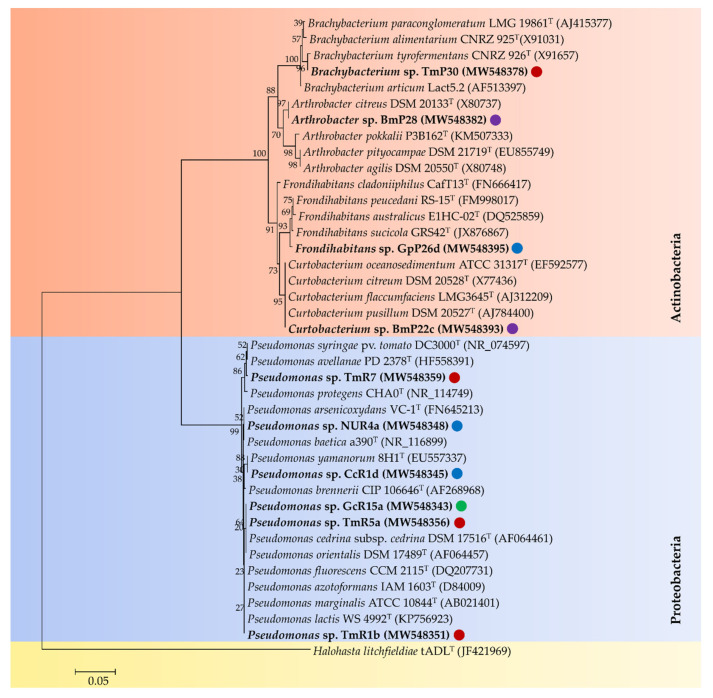
Phylogenetic tree of the 10 selected psychrotolerant strains for the formation of potential bacterial consortia. The dendrogram was constructed using the Neighbor-Joining method based on the partial sequence of the 16S rRNA gene. The tree has arbitrarily been rooted by the Antarctic archaeon *Halohasta litchfieldiae*. Values of 1000 bootstraps are informed at the branching point. GenBank accession numbers of 16S rRNA sequences are indicated in parentheses. Scale bar represents 0.05 substitutions per nucleotide positions. The circles represent the regions of Chile. Red circles, Valparaíso Region; Green circles, Libertador General Bernardo O’Higgins Region; blue circles, Ñuble Region; and purple circle, Magallanes and Chilean Antarctica Region.

The antimicrobial activities against the bacterial phytopathogens *Pseudomonas syringae* pv. *syringae* Cc1 (*Pss*), *Pectobacterium carotovorum* NCPPB 312 (*Pc*), *Clavibacter michiganensis* subsp. *michiganensis* OP3 (*Cmm*), and *Agrobacterium tumefaciens* C58C1 (*At*) were evaluated for the 21 selected strains. Ten isolates showed activity against *Pss*, eight against *Pc*, five against *At*, and 14 against *Cmm* (Table 4). Ten strains that presented antimicrobial activity against INA bacteria (*Pss* and *Pc*) were selected for the following studies. Six selected strains belong to *Pseudomonas* genus (TmR1b, TmR5a, GcR15a, NUR4a, TmR7, and CcR1d), whereas four strains are member of the Actinobacteria phylum (*Arthrobacter* sp. BmP28, *Curtobacterium* sp. BmP22c, *Brachybacterium* sp. TmP30, and *Frondihabitans* sp. GpP26d) (Figure 4).

### 3.7. Compatibility Tests and Formulation of Bacterial Consortia

**Figure 5:**
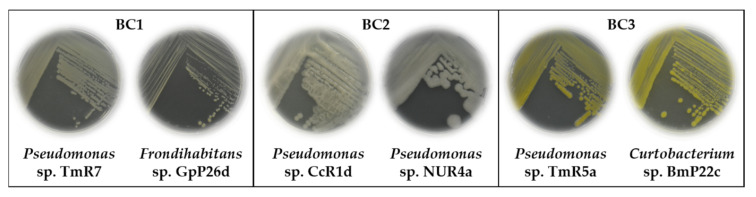
Selected bacterial consortia. The three bacterial consortia formulated with two bacteria are illustrated. Bacteria were grown in Tryptic Soy Agar (TSA) medium at room temperature. Abbreviation: BC, bacterial consortium.

The compatibilities between the ten selected strains are shown in Table 5. Thirteen potential bacterial consortia were formulated based on the compatibility assays and the previous experimental analyses. Three bacterial consortia were selected based on the presence of all the analyzed activities. Bacterial consortia (BC) compositions were BC1: *Pseudomonas* sp. TmR7 and *Frondihabitans* sp. GpP26d, BC2: *Pseudomonas* sp. CcR1d and *Pseudomonas* sp. NUR4a, and BC3: *Pseudomonas* sp. TmR5a and *Curtobacterium* sp. BmP22c (Figure 5). The characteristics of the three bacterial consortia are summarized in Table 6. 

### 3.8. Plant Growth–Promotion on Tomato Plants

**Figure 6:**
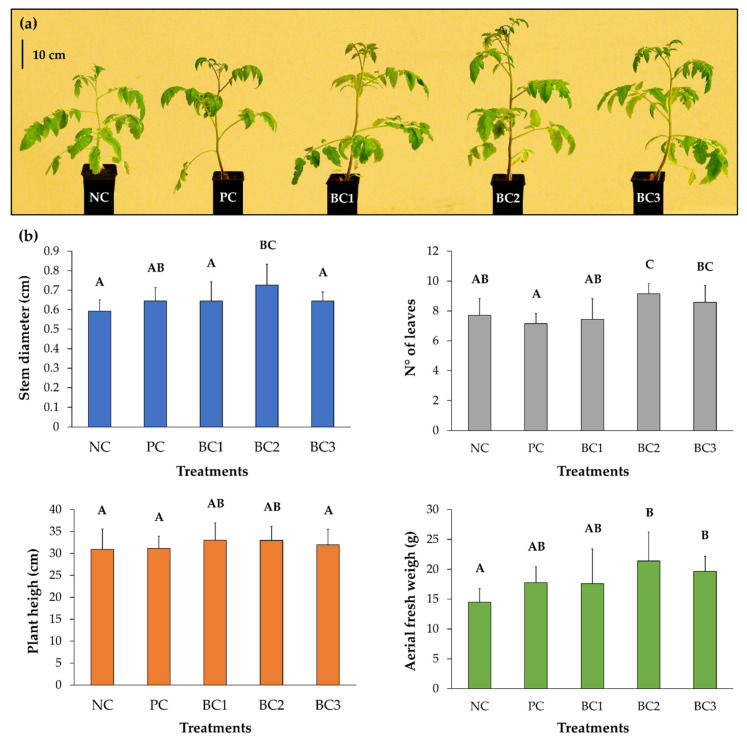
Effects of selected bacterial consortia on tomato plant growth. Tomato seedlings were arranged in peat pots and inoculated every 2 weeks in roots and at the leaves. (**a**) Representative photograph of tomato plants assay. (**b**) Graphs of parameters evaluated for tomato plants growth. Each value is a mean ± SD of seven independent replicates. Significant differences were analyzed by one-way ANOVA followed by LSD Fisher test. Means with different letters indicate significant differences (*p* < 0.05). Abbreviations: NC, negative control; PC, positive control (Nutrisac); BC1-3, bacterial consortium 1, 2, and 3, respectively.

The plant growth–promotion on tomato plants by the three selected consortia were analyzed. After BC2 treatment, plants presented an average stem diameter thickness of 0.73 cm, which was significantly higher than negative control (NC) with 0.59 cm (Figure 6). After the BC2 treatment, an average of 9.14 leaves were observed, which were significantly higher than those obtained by the NC (7.71) and the positive control (PC) (7.14). After the BC2 and BC3 treatments, a significantly higher aerial fresh weight was observed compared to the NC (14.47 g), with values of 21.39 g and 19.64 g, respectively (Figure 6).

### 3.9. Plant Growth–Promotion on Tomato Seeds

**Figure 7:**
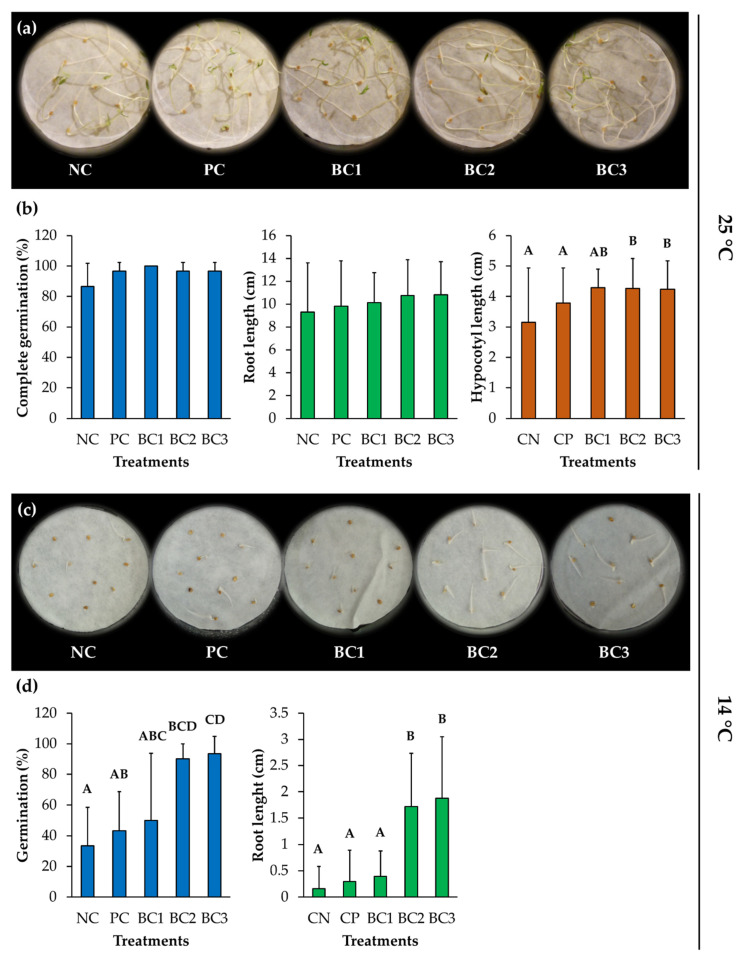
Effect of selected bacterial consortia on the growth of tomato seeds at 25 and 14 °C in the biopriming assay. (**a**) Representative photograph of tomato seed growth inoculated with selected consortia at 25 °C. (**b**) Graphs of evaluated parameters of tomato seeds growth at 25 °C. (**c**) Representative photograph of tomato seed growth inoculated with selected consortia at 14 °C. (**d**) Graphs of evaluated parameters of tomato seeds growth at 14 °C. Each value is a mean ± SD of three independent replicates, with a total of 30 seeds per treatment. Significant differences were analyzed by the Kruskal–Wallis test followed by all pairwise multiple comparisons. Means with different letters indicate significant differences (*p* < 0.05). Abbreviations: NC, negative control; PC, positive control (Nutrisac); BC1-3, bacterial consortium 1, 2, and 3, respectively.

The plant growth–promotion on tomato seeds by the three selected consortia were analyzed at 25 and 14 °C. For treatments at 25 °C, BC2 and BC3 treatments presented a significant increase in hypocotyl length, with values of 4.26 and 4.24 cm, respectively, compared to the NC (3.15 cm) (Figure 7a,b). The treatments did not show significant differences in complete germination % and root length.

For treatments at 14 °C, the germination % and the root length were analyzed (Figure 7c,d). The BC2 and BC3 treatments showed a significant increase in germination, with values of 90 and 93.3%, respectively, compared to NC (33.3%) and PC (43.3%). The root length also showed a significant increase with BC2 and BC3 treatments, with values of 1.72 and 1.88 cm, respectively, compared to NC (0.16 cm) and PC (0.3 cm).

The BC1 (*Pseudomonas* sp. TmR7 & *Frondihabitans* sp. GpP26d) did not show any plant growth–promoting effect in these assays and, therefore, was not used for the following studies.

### 3.10. Selection of One Bacterial Consortium

**Figure 8:**
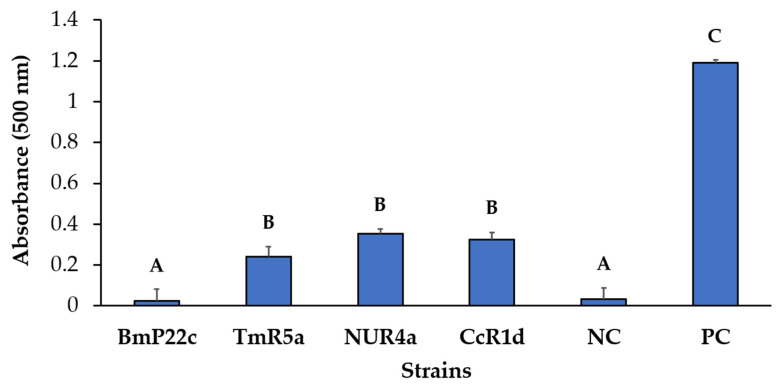
IRI activity of bacterial strains. The protein extracts were exposed to −6 °C for 48 h to measure their absorbance at 500 nm. Each value is a mean ± SD of 6 independent replicates. Significant differences were analyzed by one-way ANOVA followed by the LSD Fisher test. Means with different letters indicate significant differences (*p* < 0.05). Abbreviations: NC, negative control (*E. coli* JM109); PC, positive control (Type III AFP); IRI, ice recrystallization inhibition.

The ice recrystallization inhibition (IRI) activity and the ACC deaminase activity were evaluated in strains that composed the bacterial consortia, to select one bacterial consortium for future cold stress analyses. To determine the antifreeze potential of the bacteria, the IRI activity was evaluated, subjecting protein extracts to temperatures below zero degrees Celsius (Figure 8). *Pseudomonas* sp. strains NUR4a, CcR1d, and TmR5a presented IRI activity, with values of 0.35; 0.32; and 0.24 absorbance units, respectively, which are significantly higher compared to the negative control (0.032) (*E. coli* JM109).

**Figure 9:**
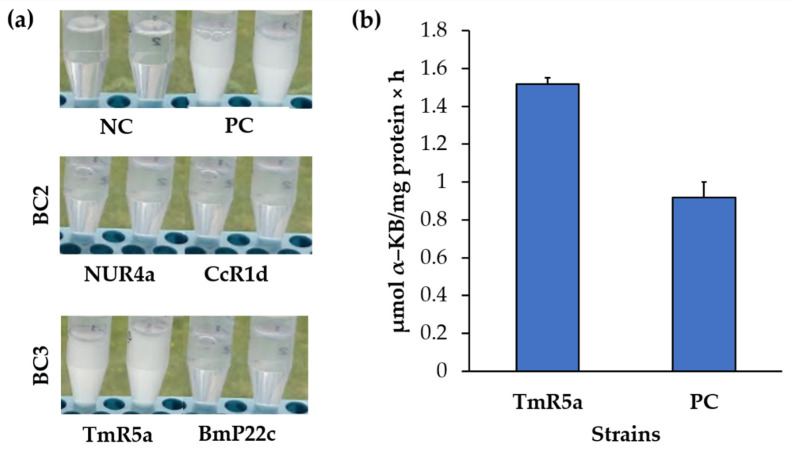
Growth in ACC and ACC deaminase activity of bacterial strains. (**a**) Growth in DF medium with ACC addition as sole carbon and nitrogen source. (**b**) ACC deaminase activity in µmol α-KB mg protein^−1^ h^−1^ of strains with positive growth. Each value is a mean ± SD of three independent replicates. Abbreviations: NC, negative control; PC, positive control (*Achromobacter* sp. 188); BC, bacterial consortium; ACC, 1-aminocyclopropane-1-carboxylic acid; α-KB, α-ketobutyrate.

To study the ACC deaminase activity, the growth of the four strains belonging to the selected bacterial consortia (BC2 and BC3) was evaluated in DF medium with ACC as sole carbon and nitrogen source (Figure 9a). Only *Pseudomonas* sp. TmR5a (BC3) showed growth under these conditions. Strain TmR5a presented an ACC deaminase activity of 1.516 µmol α-KB mg protein^−1^ h^−1^ (Figure 9b).

Therefore, BC3 (*Pseudomonas* sp. TmR5a & *Curtobacterium* sp. BmP22c) was selected because it presented all the evaluated activities together.

## 4. Discussion

In the present study, for the isolation of psychrotolerant bacteria, rhizosphere and phyllosphere of 11 wild plants were collected from three areas of the Andes Mountains and one area of Patagonia during winter of the year 2015. In this study, 130 psychrotolerant bacterial isolates were characterized, 79% from the rhizosphere and 21% from the phyllosphere (Table 2). A higher number of strains were isolated from the rhizosphere than from the phyllosphere in 9 of the 11 wild plants studied. More strains from phyllosphere than from rhizosphere were isolated only from the *Berberis* sp. plant. Several reports point to a higher richness and microbial diversity in the rhizosphere than the phyllosphere of plants [41,64,65,66]. This bacterial diversity is correlated with the total community size. The rhizosphere and the phyllosphere present different sizes of the total bacterial population. In the phyllosphere, the bacterial abundance is estimated to be approximately 10^6^ cells g^−1^, while the number of bacteria in the rhizosphere can reach up to 10^8^ cells g^−1^ dry weight [65]. Notably, this is the first study of bacteria associated with *Calycera*, *Orites*, and *Chusquea* plant genera. Previous reports highlight the isolation of bacteria with plant growth-promoting properties from *Thlaspi*, *Baccharis*, *Nothofagus*, *Haplopappus*, *Gnaphalium*, and *Gaultheria* [67,68,69,70,71,72,73]. In contrast, only pathogenic bacteria have been associated with *Berberis* [74]. In our study, 6 of the 11 wild plants presented zero or very low number of isolates from the phyllosphere (Figure 2). Under the conditions carried out in this study, the detection limit was 10^3^ CFU mL^−1^ (1 CFU on the plate), obtaining a number <10^3^ CFU mL^−1^ (no isolates on the plate) for *Baccharis* sp., *Nothofagus* sp., *Orites* sp., and *Chusquea* sp., while 2 × 10^3^ CFU mL^−1^ (2 isolates) were obtained for *Nothofagus* sp. in association with the lichen *Usnea* sp., *Haplopappus* sp., and *Gaultheria* sp. The low bacterial number can be partly explained due to the antimicrobial activities of some of these wild plants and the lichen [75,76,77,78,79,80,81,82], and the low recovery rates of culturable bacteria from the rhizosphere and phyllosphere of plants [83,84] under the conditions used in this study.

Seventy of the 130 bacterial isolates that presented a value ≥33% BCS were further characterized. Interestingly, the Valparaíso Region presented the highest number of psychrotolerant strains (55.7%). These bacteria were isolated from Paso Internacional Los Libertadores (Portillo, Valparaíso Region, Chile), which was the highest altitude wild flora sampling site (3149 m.a.s.l.) and presents a winter (from April to October) with temperatures below 0 °C, reaching −20 °C (http://www.gobernacionlosandes.gov.cl/inicio/ (accessed on 5 February 2021)). The identification by the 16S rRNA gene sequence analysis indicated that these 70 strains belong to 18 different bacterial genera of four bacterial phyla (Figure 2). In our study, Proteobacteria, Actinobacteria, Firmicutes, and Bacteroidetes are among the most abundant bacterial phyla in the rhizosphere and the phyllosphere, in accordance with previous reports [65,66]. Different isolates of *Pseudomonas*, *Stenotrophomonas*, *Serratia*, *Brevundimonas*, *Arthrobacter*, *Pseudarthrobacter*, *Paenarthrobacter*, *Curtobacterium*, *Brachybacterium*, *Sporosarcina*, *Bacillus*, *Solibacillus*, *Janthinobacterium*, *Flavobacterium*, and *Pedobacter* described in the rhizosphere and the phyllosphere of plants in the present study, have been reported previously in plants and also in soil, water, ice, and animals [85,86,87,88,89,90,91,92,93,94,95]. Interestingly, *Frondihabitans* sp. GpP26d was isolated from the phyllosphere of a *Gaultheria* plant. Members of the *Frondihabitans* genus has only been associated with plants and lichens [96,97,98,99,100]. On the other hand, some members of the *Xanthomonas* and *Clavibacter* genera are phytopathogens, which are in the rhizosphere, the phyllosphere, and the endosphere of different plants [44,63,101,102]. In the present study, the bacterial strains that are closely related to phytopathogens, *Xanthomonas* sp. HsR18 and *Clavibacter* sp. BmP21 that were isolated from the rhizosphere of *Haplopappus* sp. and the phyllosphere of *Berberis* sp., respectively, were discarded for the formulation of bacterial consortia. Except for the *Xanthomonas* and *Clavibacter* genera, the psychrotolerance of all the bacterial genera analyzed in this study have been reported [21,24,25,100,103,104,105,106,107].

In the present study, *Pseudomonas* and *Stenotrophomonas* genera showed the highest number of psychrotolerant strains, with 18 and 17 isolates, respectively (Figure 2). Strains of the *Pseudomonas* genus were isolated in the rhizosphere (16) and the phyllosphere (2). *Stenotrophomonas* strains genus were also isolated from the rhizosphere (12) and the phyllosphere (5). *Pseudomonas* is the genus of Gram-negative bacteria with the largest number of recognized species, where more than 220 species have been characterized [108]. The ubiquity and metabolic versatility of this genus allow it to colonize a wide range of natural habitats and adopt a variety of lifestyles. *Pseudomonas* strains have been isolated from each of the ecological niches within plants including roots (rhizosphere), leaves (phyllosphere), and tissues (endosphere) [108,109]. In previous studies, *Pseudomonas* strains isolated from Chilean agricultural soils with nematicidal and herbicide-degrading activities have been characterized [110,111]. On the other hand, the *Stenotrophomonas* genus has been associated with soil and plants [112,113,114], showing beneficial effects on plant growth and health [86]. In our study, all the *Stenotrophomonas* strains isolated from the four regions were closely related to *Stenotrophomonas rhizophila* species. *Stenotrophomonas rhizophila* is a rhizosphere-associated species that display antifungal properties [115].

The search for psychrotolerant PGPB that can exert their role of plant growth-promoting at low temperatures is of high importance for agriculture [12,105,106]. The benefits of mixed PGPB inoculation are due to the combination of plant growth-promoting (PGP) activities [16]. In this context, for the selection of a group of psychrotolerant bacterial consortia, different PGP activities were evaluated in the 70 selected psychrotolerant strains (Figure 3, Table 3). In addition, antimicrobial activities against well-known phytopathogenic and ice nucleation active bacteria (Table 4), such as *Pseudomonas syringae* and *Pectobacterium carotovorum* that magnify the stress caused by cold in plants [2,116], *Agrobacterium tumefaciens* that infects a wide range of plants and causes plant tumors called crown galls [117], and *Clavibacter michiganensis* subsp. *michiganensis,* which is an important tomato phytopathogen [44,63,101], were analyzed. These PGP activities have also been used to select bacterial consortia for plant growth–promotion under stress (cold, drought, salinity) and non-stressful conditions [28,42,118,119,120,121,122].

The plant growth–promotion by three consortia of pairs of bacterial strains (Figure 5 and Table 6) were studied. Interestingly, two bacterial consortia (BC2 and BC3) promoted the growth of tomato plants at 25 °C (Figure 6) and the germination of tomato seeds at 14 °C (Figure 7), demonstrating the potential of these consortia to promote plant growth at low temperatures. In the present study, the bacterial consortia composed of *Pseudomonas* sp. CcR1d & *Pseudomonas* sp. NUR4a (BC2) and *Pseudomonas* sp. TmR5a & *Curtobacterium* sp. BmP22c (BC3), after 12 days at 14 °C, promoted the germination of tomato seeds by ≥90% compared to 33% control germination, and significantly increased the root lengths (Figure 7). Subramanian et al. [25] reported between 90 and 100% germination of tomato seeds when treated with strains of *Pseudomonas* and *Flavobacterium* after 10 days at 15 °C, while the control germination was 45%. Mishra et al. [22] reported the promotion of wheat seed germination (85%) by *Pseudomonas* sp. NARs9 compared to 71% control germination, after 7 days at 18 °C. Yarzábal et al. [26] showed an increase of around 32% in the length of roots of wheat seeds treated with the Antarctic *Pseudomonas* sp. CIBEA71 compared to the control treatment, after 5 days at 16 °C. Zubair et al. [27] reported that the treatment with *Bacillus* strains increases the vigor index of wheat seeds after 7 days at 14 °C.

*Pseudomonas* strains TmR5a, NUR4a, and CcR1d showed IRI activity (Figure 8), which could contribute to plant adaptation and protection against cold stress. Various microorganisms that survive and proliferate under freezing temperatures possess the ice-binding proteins, such as antifreeze proteins (AFPs), which regulate the formation and growth of ice crystals [41,123]. The AFPs act by binding to ice crystals to induce IRI, preventing the generation of large ice crystals [124]. In addition, *Pseudomonas* sp. strains TmR5a and NUR4a, and *Curtobacterium* sp. BmP22c possess the *acdS* gene that encodes ACC deaminase. *Pseudomonas* sp. TmR5a showed an ACC deaminase activity of 1.516 µmol α-KB mg protein^−1^ h^−1^ (Figure 9), which exceeds the value of ~20 nmol α-KB mg protein^−1^ h^−1^ that allowed a bacterium to grow in ACC and act as a PGPB [58]. Based on its ACC deaminase activity, *Pseudomonas* sp. TmR5a has the potential to slow down the production of ethylene induced by abiotic stress and its associated adverse effects on plants [121]. In contrast, no ACC deaminase activity was observed in strains NUR4a and BmP22c under these conditions.

In our study, the psychrotolerant bacterial mixture formulated with *Pseudomonas* sp. TmR5a and *Curtobacterium* sp. BmP22c (BC3) was selected as the most promising consortium due to its plant protective and growth-promoting activities. The BC3 is composed of strains from the rhizosphere of *Thlaspi* and the phyllosphere of *Berberis* (Figure 1). This is the first study that reports beneficial bacteria from a plant of the *Berberis* genus. *Pseudomonas* strains have competitive advantages compared to diverse other microorganisms due to their plant growth-promoting traits under normal, as well as stressful environmental conditions [125]. *Pseudomonas* strains have been reported in growth–promotion of canola, wheat, and tomato under cold stress [17,18,19,22,25,126]. On the other hand, the cosmopolitan *Curtobacterium* strains are related mainly with the phyllosphere and have been associated with plant growth–promotion under normal conditions [127,128,129], protection against phytopathogens such as *Pseudomonas syringae* [130], and alleviation of salinity stress in plants [131,132,133]. However, the alleviation of cold stress by *Curtobacterium* has not been reported before. This is the first report in which a bacterial consortium is formulated only with strains of *Pseudomonas* and *Curtobacterium* genera. Other studies reported bacterial consortia composed of *Pseudomonas*, *Curtobacterium* and other genera (containing from 3 to 12 strains) for the germination of cotton seeds [134], the protection of tomato plants against the phytopathogen *Pseudomonas syringae* pv. *tomato* [135], and the bioremediation of petroleum [136]. In addition, bacteria of the *Pseudomonas* and *Curtobacterium* genera are the most abundant in ecosystems related to poplar, willow, grapevine, rice, and nectarine plants [137,138,139,140], showing a close relationship to diverse plants. Bacterial consortia to alleviate the cold stress in plants have been scarcely reported. The bacterial consortium composed of *Bacillus amyloliquefaciens* Bk7 and *Brevibacillus laterosporus* B4, which solubilizes phosphates and produces IAA and siderophores, protects and improves the growth of rice under cold stress [28]. The rice seedlings inoculated with this consortium 1 week before the chilling stress (5 °C for 24 h) improves survival rate, plant height, and shoot number. The bacterial consortium of *Bacillus cereus* AR156, *Bacillus subtilis* SM21, and *Serratia* sp. XY21, which produces IAA and has antagonistic activity against phytopathogenic INA bacterium *P. syringae*, fungi, and oomycetes, improves the survival rate of tomato seedlings subjected to chilling stress (4 °C for 7 days) [29].

The selected bacterial consortium formulated with *Pseudomonas* sp. TmR5a and *Curtobacterium* sp. BmP22c, which exhibits several plant growth-promoting and protective activities including plant growth-promoting under chilling stress (<15 °C), is an attractive candidate for future applications in agriculture. Additional assays will be carried out to study its protective effect on plants under freezing stress, which are required prior to its evaluation on the field against frost.

## 5. Conclusions

In this study, seventy highly psychrotolerant bacteria with plant growth-promoting activities of one hundred thirty isolates from wild flora from the Andes Mountains and Patagonia of Chile were characterized. These bacteria belong to Proteobacteria, Actinobacteria, Firmicutes, and Bacteroidetes phyla, and 18 genera. Notably, this is the first report of bacteria associated with *Calycera*, *Orites*, and *Chusquea* plants; one of the *Chusquea* strains is a member of BC2. Two bacterial consortia: *Pseudomonas* sp. CcR1d & *Pseudomonas* sp. NUR4a (BC2) and *Pseudomonas* sp. TmR5a & *Curtobacterium* sp. BmP22c (BC3) presented auxin production, phosphate solubilization activity, *nifH* and *acdS* genes, antimicrobial activities against important phytopathogens, and growth promotion of tomato plants and seeds under normal and cold stress conditions. Due to its additional anti-stress ACC deaminase and IRI activities, the bacterial consortium composed of *Pseudomonas* sp. TmR5a and *Curtobacterium* sp. BmP22c is an attractive candidate for genome analyses, evaluation of its epiphytic and rhizosphere colonization potential, and characterization of its plant protection against frost. A bioproduct based on this psychrotolerant bacterial consortium is an eco-friendly alternative for the protection of agricultural crops to cold stress and, in addition, to phytopathogens.

## Figures and Tables

**Table 1:** Primers used in this study.

Primer	Sequence (5’-3’)	Gene Target	Size (pb)	Reference
27F	AGAGTTTGATCMTGGCTCAG	16S ARNr	1465	[35]
1492R	TACGGYTACCTTGTTACGACTT			
nifH-F-Rösch	AAAGGYGGWATCGGYAARTCCACCA	*nifH*	458	[36]
nifH-R-Rösch	TTGTTSGCSGCRTACATSGCCATCAT			
DegACC-F	GGBGGVAAYAARMYVMGSAAGCTYGA	*acdS*	750	[37]
DegACC-R	TTDCCHKYRTANACBGGRTC			

**Table 2:** Number of bacterial isolates from wild plants.

	Number of Isolates													
	Rhizosphere							Phyllosphere						
Wild plant (Code for Isolates)	Total	Id	L	M	H	VH	E	Total	Id	L	M	H	VH	E
*Calycera* sp. (Cp)	16	11	0	5	0	4	7	3	3	0	0	1	0	2
*Thlaspi* sp. (Tm)	17	13	3	1	4	7	2	10	8	0	2	2	2	4
*Haplopappus* sp. (Hs)	9	3	0	6	1	0	2	2	1	1	0	0	1	0
*Baccharis* sp. (Bl)	10	3	1	6	0	3	0	0	0	0	0	0	0	0
*Gnaphalium* sp. (Gc)	18	9	3	6	4	2	3	2	2	0	0	1	0	1
*Gaultheria* sp. (Gp)	8	1	1	6	0	1	0	2	1	1	0	0	0	1
*Nothofagus* sp. + *Usnea* sp. (NU)	7	2	0	5	1	1	0	1	0	1	0	0	0	0
*Orites* sp. (Om)	3	1	1	1	0	1	0	0	0	0	0	0	0	0
*Chusquea* sp. (Cc)	10	3	1	6	2	0	1	1	0	0	1	0	0	0
*Berberis* sp. (Bm)	5	3	0	2	1	0	2	6	6	0	0	1	2	3
*Nothofagus* sp. (Nd)	0	0	0	0	0	0	0	0	0	0	0	0	0	0
Total isolates	**103**	**49**	**10**	**44**	**13**	**19**	**17**	**27**	**21**	**3**	**3**	**5**	**5**	**11**

Id, Identified isolates showing ≥33% bacterial cell survival (BCS); L, low (<10% BCS); M, moderate (10–32% BCS); H, high (33–55% BCS); VH, very high (56–78% BCS); E, excellent (79–100% BCS).

**Table 3:** Selected strains showing the higher phosphate solubilization activity and presence of *nifH* and *acdS* genes revealed by PCR.

Strains	PS at RT/PS at 4 °C	*nifH*/ *acdS*	Strains	PS at RT/ 4 °C	*nifH*/ *acdS*
*Pseudomonas* sp. TmR1b *	+/+	+/−	*Stenotrophomonas* sp. TmP43c *	−/−	+/+
*Pseudomonas* sp. TmR34a	+/+	+/−	*Stenotrophomonas* sp. TmR19b	−/−	+/−
*Pseudomonas* sp. TmR35a	+/+	+/−	*Brevundimonas* sp. CpR34a	−/−	+/−
*Pseudomonas* sp. TmR5a *	+/+	+/+	*Arthrobacter* sp. BmP28 *	+/−	+/+
*Pseudomonas* sp. GcR15a *	−/−	+/+	*Arthrobacter* sp. BmR37	+/−	+/−
*Pseudomonas* sp. NUR4a *	+/+	−/+	*Arthrobacter* sp. CpR32a	−/−	+/−
*Pseudomonas* sp. BlR7b *	+/+	+/+	*Paenarthrobacter* sp. BlR11a	−/−	+/−
*Pseudomonas* sp. BlR3 *	+/+	+/+	*Pseudarthrobacter* sp. TmP42	−/−	+/−
*Pseudomonas* sp. TmR7 *	+/+	+/+	*Pseudarthrobacter* sp. GcR10	−/−	+/−
*Pseudomonas* sp. CcR1d *	+/+	+/−	*Curtobacterium* sp. GcR11a	−/−	+/−
*Serratia* sp. CpR7a	+/+	−/−	*Curtobacterium* sp. BmP22c *	−/−	−/+
*Serratia* sp. TmR3a *	+/+	+/−	*Curtobacterium* sp. BmP18c *	−/−	−/−
*Stenotrophomonas* sp. BmP26c	−/−	+/−	*Brachybacterium* sp. TmP24d	−/−	+/+
*Stenotrophomonas* sp. BmR35 *	−/−	+/+	*Brachybacterium* sp. TmP30	−/−	+/+
*Stenotrophomonas* sp. CpP23d *	−/−	+/+	*Frondihabitans* sp. GpP26d *	−/−	+/+
*Stenotrophomonas* sp. CpR10 *	−/−	+/+	*Sporosarcina* sp. HsP12b *	−/−	+/+
*Stenotrophomonas* sp. CpR13a	−/−	+/+	*Sporosarcina* sp. HsR8 *	−/−	+/+
*Stenotrophomonas* sp. CpR8a	−/−	+/+	*Bacillus* sp. GcP2b	+/−	−/−
*Stenotrophomonas* sp. CpR12a	−/−	+/−	*Pedobacter* sp. CpR35b	−/−	−/−
*Stenotrophomonas* sp. GpR8b	−/−	+/+	*Flavobacterium* sp. CpR5c *	+/+	+/−
*Stenotrophomonas* sp. GcR23 *	−/−	+/+			

* Selected strains. +, positive activity/amplification; −, negative activity/amplification; PS, phosphate solubilization; RT, room temperature.

**Table 4:** Antagonistic activity against phytopathogenic bacteria by the radial streak method.

Strains	*Pss*			*Pc*			*Cmm*		*At*	
	YM	MH	KB	YM	MH	KB	YM	MH	YM	MH
*Pseudomonas* sp. TmR1b *	+	−	++	+	−	+/−	+	++	−	−
*Pseudomonas* sp. TmR5a *	−	−	+	−	−	−	+	+	−	−
*Pseudomonas* sp. GcR15a *	+/−	−	+	+	−	+/−	++	+	−	−
*Pseudomonas* sp. NUR4a *	++	−	+	++	−	−	++	++	+	−
*Pseudomonas* sp. BlR7b	−	−	−	−	−	−	−	−	−	−
*Pseudomonas* sp. BlR3	−	−	−	−	−	−	−	−	−	−
*Pseudomonas* sp. TmR7 *	−	−	−	+/−	−	−	+/−	+/−	−	−
*Pseudomonas* sp. CcR1d *	−	−	+	−	−	+/−	++	++	+	−
*Serratia* sp. TmR3a	−	−	−	−	−	−	+	−	−	−
*Stenotrophomonas* sp. BmR35	−	−	−	−	−	−	−	−	−	−
*Stenotrophomonas* sp. CpP23d	−	−	−	−	−	−	−	+	−	−
*Stenotrophomonas* sp. CpR10	−	−	−	−	−	−	−	−	−	−
*Stenotrophomonas* sp. GcR23	−	−	−	−	−	−	−	−	−	−
*Stenotrophomonas* sp. TmP43c	−	−	−	−	−	−	−	−	−	−
*Arthrobacter* sp. BmP28 *	+	−	+	+	−	+	++	−	++	−
*Curtobacterium* sp. BmP22c *	+	−	−	++	−	−	++	+/−	++	−
*Brachybacterium* sp. TmP30 *	−	+	−	−	−	−	−	+/−	−	−
*Frondihabitans* sp. GpP26d *	+	−	−	+	−	−	++	+	+	+
*Sporosarcina* sp. HsP12b	−	−	−	−	−	−	−	−	−	−
*Sporosarcina* sp. HsR8	−	−	−	−	−	−	+/−	+/−	−	−
*Flavobacterium* sp. CpR5c	−	−	−	−	−	−	−	+/−	−	−
*Pseudomonas protegens* CHA0	+	−	+	++	−	+	++	+	+	−

* Selected strains. −, no inhibition; +/−, attenuated growth; +, <50% growth inhibition; ++, ≥50% growth inhibition. *Pss, Pseudomonas syringae* pv. *syringae*; *Pc, Pectobacterium carotovorum*; *Cmm, Clavibacter michiganensis* subsp. *michiganensis*; *At, Agrobacterium tumefaciens*; YM, Yeast Malt; MH, Müller-Hinton; KB, King B.

**Table 5:** Compatibility between selected bacterial strains.

Strains	TmR1b	TmR5a	GcR15a	NUR4a	TmR7	CcR1d	BmP28	BmP22c	TmP30	GpP26d
TmR1b		+	+	−	−	−	−	+	−	−
TmR5a	+		−	−	−	−	−	+	−	−
GcR15a	+	−		−	−	−	−	−	−	−
NUR4a	−	−	−		−	+	−	−	−	−
TmR7	−	−	−	−		−	+	+	−	+
CcR1d	−	−	−	+	−		−	−	−	−
BmP28	−	−	−	−	+	−		−	+	+
BmP22c	+	+	−	−	+	−	−		+	−
TmP30	−	−	−	−	−	−	+	+		−
GpP26d	−	−	−	−	+	−	+	−	−	

−, not compatible (antimicrobial activity); +, compatible (without antimicrobial activity).

**Table 6:** Characteristics of the selected bacterial consortia.

N°	Bacterial Consortia	PT Potential	Plant Growth-Promoting Activities/Determinations				Antimicrobial Activities			
		BCS%	[Auxin] */ OD_600nm_	PS at RT/ PS at 4 °C	*nifH*	*acdS*	*Pss*	*Pc*	*Cmm*	*At*
BC1	*Pseudomonas* sp. TmR7	100	0.91	+/+	+	+	−	+	+	−
	*Frondihabitans* sp. GpP26d	93.26	5.146	−/−	+	+	+	+	+	+
BC2	*Pseudomonas* sp. CcR1d	78.88	0.074	+/+	+	−	+	+	+	+
	*Pseudomonas* sp. NUR4a	70.55	1.342	+/+	−	+	+	+	+	+
BC3	*Pseudomonas* sp. TmR5a	56.23	1.223	+/+	+	+	+	−	+	−
	*Curtobacterium* sp. BmP22c	84.5	1.37	−/−	−	+	+	+	+	+

* Auxin concentration (µg mL^−1^); BC, bacterial consortium; PT, psychrotolerant; BCS%, bacterial cell survival percentage; PS, phosphate solubilization; RT, room temperature; *Pss, Pseudomonas syringae* pv. *syringae*; *Pc, Pectobacterium carotovorum*; *Cmm, Clavibacter michiganensis* subsp. *michiganensis*; *At, Agrobacterium tumefaciens*; +, positive activity/determination; −, negative activity/determination.

## Data Availability

The partial 16S rRNA gene sequences of the bacteria were deposited in GenBank under the accession numbers MW548335-MW548404.

## References

[1] Wang H., Wang H., Shao H., Tang X. (2016). Recent advances in utilizing transcription factors to improve plant abiotic stress tolerance by transgenic technology. Front. Plant Sci..

[2] FAO (2005). Frost Protection: Fundamentals, Practice and Economics.

[3] Duman J.G., Wisniewski M. (2014). The use of antifreeze proteins for frost protection in sensitive crop plants. Environ. Exp. Bot..

[4] Raza A., Razzaq A., Mehmood S.S., Zou X., Zhang X., Lv Y., Xu J. (2019). Impact of climate change on crops adaptation and strategies to tackle its outcome: A review. Plants.

[5] Liu Y., Dang P., Liu L., He C. (2019). Cold acclimation by the CBF–COR pathway in a changing climate: Lessons from *Arabidopsis thaliana*. Plant Cell Rep..

[6] Barlow K.M., Christy B.P., O’Leary G.L., Riffkin P.A., Nuttall J.G. (2015). Simulating the impact of extreme heat and frost events on wheat crop production: A review. Field Crops Res..

[7] Bagati S., Mahajan R., Nazir M., Dar A.A., Zargar S.M., Zargar S., Zargar M. (2018). “Omics”: A gateway towards abiotic stress tolerance. Abiotic Stress-mediated Sensing and Signaling in Plants: An Omics Perspective.

[8] Glick B.R. (2012). Plant growth-promoting bacteria: Mechanisms and applications. Scientifica.

[9] Rilling J.I., Acuña J.J., Sadowsky M.J., Jorquera M.A. (2018). Putative nitrogen-fixing bacteria associated with the rhizosphere and root endosphere of wheat plants grown in an andisol from Southern Chile. Front. Microbiol..

[10] Bakhshandeh E., Gholamhosseini M., Yaghoubian Y., Pirdashti H. (2020). Plant growth promoting microorganisms can improve germination, seedling growth and potassium uptake of soybean under drought and salt stress. Plant Growth Regul..

[11] Velásquez A., Vega-Celedón P., Fiaschi G., Agnolucci M., Avio L., Giovannetti M., D’Onofrio C., Seeger M. (2020). Responses of *Vitis vinifera* cv. Cabernet Sauvignon roots to the arbuscular mycorrhizal fungus *Funneliformis mosseae* and the plant growth-promoting rhizobacterium *Ensifer meliloti* include changes in volatile organic compounds. Mycorrhiza.

[12] Mishra P.K., Bisht S.C., Ruwari P., Selvakumar G., Joshi G.K., Bisht J.K., Bhatt J.C., Gupta H.S. (2011). Alleviation of cold stress in inoculated wheat (*Triticum aestivum* L.) seedlings with psychrotolerant *Pseudomonas* from NW Himalayas. Arch. Microbiol..

[13] Pandey P., Bisht S., Sood A., Aeron A., Sharma G.D., Maheshwari D.K., Maheshwari D. (2012). Consortium of plant-growth-promoting bacteria: Future perspective in agriculture. Bacteria in Agrobiology: Plant Probiotics.

[14] Majeed A., Muhammad Z., Ahmad H. (2018). Plant growth promoting bacteria: Role in soil improvement, abiotic and biotic stress management of crops. Plant Cell Rep..

[15] Bradáčová K., Florea A.S., Bar-Tal A., Minz D., Yermiyahu U., Shawahna R., Kraut-Cohen J., Zolti A., Erel R., Dietel K. (2019). Microbial consortia versus single-strain inoculants: An advantage in PGPM-assisted tomato production?. Agronomy.

[16] Menéndez E., Paço A. (2020). Is the application of plant probiotic bacterial consortia always beneficial for plants? Exploring synergies between rhizobial and non-rhizobial bacteria and their effects on agro-economically valuable crops. Life.

[17] Sun X., Griffith M., Pasternak J.J., Glick B.R. (1995). Low temperature growth, freezing survival and production of antifreeze protein by the plant growth promoting rhizobacterium *Pseudomonas putida* GR12-2. Can. J. Microbiol..

[18] Cheng Z., Park E., Glick B.R. (2007). 1-Aminocyclopropane-1-carboxylate deaminase from *Pseudomonas putida* UW4 facilitates the growth of canola in the presence of salt. Can. J. Microbiol..

[19] Mishra P.K., Mishra S., Selvakumar G., Bisht S.C., Bisht J.K., Kundu S., Gupta H.S. (2008). Characterization of a psychrotolerant plant growth promoting *Pseudomonas* sp. strain PGERs17 (MTCC 9000) isolated from North Western Indian Himalayas. Ann. Microbiol..

[20] Selvakumar G., Kundu S., Joshi P., Gupta A.D., Nazim S., Mishra P.K., Gupta H.S. (2008). Characterization of a cold-tolerant plant growth-promoting bacterium *Pantoea dispersa* 1A isolated from a sub-alpine soil in the North Indian Himalayas. World J. Microbiol. Biotechnol..

[21] Selvakumar G., Mohan M., Kundu S., Gupta A.D., Joshi P., Nazim S., Gupta H.S. (2008). Cold tolerance and plant growth promotion potential of *Serratia marcescens* strain SRM (MTCC 8708) isolated from flowers of summer squash (*Cucurbita pepo*). Lett. Appl. Microbiol..

[22] Mishra P.K., Mishra S., Bisht S.C., Selvakumar G., Kundu S., Bisht J.K., Gupta H.S. (2009). Isolation, molecular characterization and growth-promotion activities of a cold tolerant bacterium *Pseudomonas* sp. NARs9 (MTCC9002) from the Indian Himalayas. Biol. Res..

[23] Theocharis A., Bordiec S., Fernandez O., Paquis S., Dhondt-Cordelier S., Baillieul F., Clément C., Barka E.A. (2012). *Burkholderia phytofirmans* PsJN primes *Vitis vinifera* L. and confers a better tolerance to low nonfreezing temperatures. Mol. Plant-Microbe Interact..

[24] Verma P., Yadav A.N., Khannam K.S., Panjiar N., Kumar S., Saxena A.K., Suman A. (2015). Assessment of genetic diversity and plant growth promoting attributes of psychrotolerant bacteria allied with wheat (*Triticum aestivum*) from the northern hills zone of India. Ann. Microbiol..

[25] Subramanian P., Kim K., Krishnamoorthy R., Mageswari A., Selvakumar G., Sa T. (2016). Cold stress tolerance in psychrotolerant soil bacteria and their conferred chilling resistance in tomato (*Solanum lycopersicum* Mill.) under low temperatures. PLoS ONE.

[26] Yarzábal L.A., Monserrate L., Buela L., Chica E. (2018). Antarctic *Pseudomonas* spp. promote wheat germination and growth at low temperatures. Polar Biol..

[27] Zubair M., Hanif A., Farzand A., Sheikh T.M.M., Khan A.R., Suleman M., Ayaz M., Gao X. (2019). Genetic screening and expression analysis of psychrophilic *Bacillus* spp. reveal their potential to alleviate cold stress and modulate phytohormones in wheat. Microorganisms.

[28] Kakar K.U., Ren X.L., Nawaz Z., Cui Z.Q., Li B., Xie G.L., Hassan M.A., Ali E., Sun G.C. (2016). A consortium of rhizobacterial strains and biochemical growth elicitors improve cold and drought stress tolerance in rice (*Oryza sativa* L.). Plant Biol..

[29] Wang C., Wang C., Gao Y.L., Wang Y.P., Guo J.H. (2016). A Consortium of three plant growth-promoting rhizobacterium strains acclimates *Lycopersicon esculentum* and confers a better tolerance to chilling stress. J. Plant Growth Regul..

[30] Jorquera M.A., Maruyama F., Ogram A.V., Navarrete O.U., Lagos L.M., Inostroza N.G., Acuña J.J., Rilling J.I., Mora M.L. (2016). Rhizobacterial community structures associated with native plants grown in Chilean extreme environments. Microb. Ecol..

[31] Orellana R., Macaya C., Bravo G., Dorochesi F., Cumsille A., Valencia R., Rojas C., Seeger M. (2018). Living at the frontiers of life: Extremophiles in Chile and their potential for bioremediation. Front. Microbiol..

[32] Jorquera M.A., Inostroza N.G., Lagos L.M., Barra P.J., Marileo L.G., Rilling J.I., Campos D.C., Crowley D.E., Richardson A.E., Mora M.L. (2014). Bacterial community structure and detection of putative plant growth-promoting rhizobacteria associated with plants grown in Chilean agro-ecosystems and undisturbed ecosystems. Biol. Fertil. Soils.

[33] Araya M.A., Valenzuela T., Inostroza N.G., Maruyama F., Jorquera M.A., Acuña J.J. (2020). Isolation and characterization of cold-tolerant hyper-ACC-degrading bacteria from the rhizosphere, endosphere, and phyllosphere of Antarctic vascular plants. Microorganisms.

[34] Cavieres L.A., Papic C., Castor C. (1999). Variación altitudinal en los síndromes de dispersión de semillas de la vegetación andina de la cuenca del Río Molina, Chile central (33 S). Gayana Bot..

[35] Weisburg W.G., Barns S.M., Pelletier D.A., Lane D.J. (1991). 16S ribosomal DNA amplification for phylogenetic study. J. Bacteriol..

[36] Rösch C., Mergel A., Bothe H. (2002). Biodiversity of denitrifying and dinitrogen-fixing bacteria in an acid forest soil. Appl. Environ. Microbiol..

[37] Kumar A., Kumar A., Pratush A. (2014). Molecular diversity and functional variability of environmental isolates of *Bacillus* species. Springerplus.

[38] Vega-Celedón P., Canchignia Martínez H., González M., Seeger M. (2016). Biosíntesis de ácido indol-3-acético y promoción del crecimiento de plantas por bacterias. Cultiv. Trop..

[39] Oberhänsli T., Défago G., Haas D. (1991). Indole-3-acetic acid (IAA) synthesis in the biocontrol strain CHA0 of *Pseudomonas fluorescens*: Role of tryptophan side chain oxidase. J. Gen. Microbiol..

[40] Jousset A., Schuldes J., Keel C., Maurhofer M., Daniel R., Scheu S., Thuermer A. (2014). Full-genome sequence of the plant growth-promoting bacterium *Pseudomonas protegens* CHA0. Genome Announc..

[41] Cid F.P., Inostroza N.G., Graether S.P., Bravo L.A., Jorquera M.A. (2017). Bacterial community structures and ice recrystallization inhibition activity of bacteria isolated from the phyllosphere of the Antarctic vascular plant *Deschampsia antarctica*. Polar Biol..

[42] Barra P.J., Inostroza N.G., Acuña J.J., Mora M.L., Crowley D.E., Jorquera M.A. (2016). Formulation of bacterial consortia from avocado (*Persea americana* Mill.) and their effect on growth, biomass and superoxide dismutase activity of wheat seedlings under salt stress. Appl. Soil Ecol..

[43] Osorio M.E., Quiroz K.A., Carvajal M.A., Vergara A.P., Sánchez E.Y., González C.E., Catalán K.S. (2016). Synthesis, anti-phytopathogenic and DPPH radical scavenging activities of C-prenylated acetophenones and benzaldehydes. J. Chil. Chem. Soc..

[44] Valenzuela M., Besoain X., Durand K., Cesbron S., Fuentes S., Claverías F., Jacques M.A., Seeger M. (2018). *Clavibacter michiganensis* subsp. *michiganensis* strains from Central Chile exhibit low genetic diversity and sequence types match strains in other parts of the world. Plant Pathol..

[45] Kucheryava N., Fiss M., Auling G., Kroppenstedt R.M. (1999). Isolation and characterization of epiphytic bacteria from the phyllosphere of apple, antagonistic *in vitro* to *Venturia inaequalis*, the causal agent of apple scab. Syst. Appl. Microbiol..

[46] Barrientos-Díaz L., Gidekel M., Gutiérrez-Moraga A. (2008). Characterization of rhizospheric bacteria isolated from *Deschampsia antarctica* Desv. World J. Microbiol. Biotechnol..

[47] Strahsburger E., Retamales P., Estrada J., Seeger M. (2016). Microdot method: Used with chromogenic agar is a useful procedure for sanitary monitoring in aquaculture. Lat. Am. J. Aquat. Res..

[48] Méndez V., Fuentes S., Morgante V., Hernández M., González M., Moore E., Seeger M. (2017). Novel hydrocarbonoclastic metal-tolerant *Acinetobacter* and *Pseudomonas* strains from Aconcagua river oil-polluted soil. J. Soil Sci. Plant Nutr..

[49] Bravo G., Vega-Celedón P., Gentina J.C., Seeger M. (2020). Bioremediation by *Cupriavidus metallidurans* strain MSR33 of mercury-polluted agricultural soil in a rotary drum bioreactor and its effects on nitrogen cycle microorganisms. Microorganisms.

[50] Tamura K., Peterson D., Peterson N., Stecher G., Nei M., Kumar S. (2011). MEGA5: Molecular evolutionary genetics analysis using maximum likelihood, evolutionary distance, and maximum parsimony methods. Mol. Biol. Evol..

[51] Patten C., Glick B. (2002). Role of *Pseudomonas putida* indoleacetic acid in development of the host plant root system. Appl. Environ. Microbiol..

[52] Yang S., Zhang Q., Guo J., Charkowski A., Glick B., Ibekwe A., Cooksey D., Yang C.H. (2007). Global effect of indole-3-acetic acid biosynthesis on multiple virulence factors of *Erwinia chrysanthemi* 3937. Appl. Environ. Microbiol..

[53] Vicente C.S.L., Nascimento F., Espada M., Barbosa P., Mota M., Glick B., Oliveira S. (2012). Characterization of bacteria associated with pinewood nematode *Bursaphelenchus xylophilus*. PLoS ONE.

[54] Nautiyal C.S. (1999). An efficient microbiological growth medium for screening phosphate solubilizing microorganisms. FEMS Microbiol. Lett..

[55] Coman M.M., Verdenelli M.C., Cecchini C., Silvi S., Orpianesi C., Boyko N., Cresci A. (2014). In vitro evaluation of antimicrobial activity of *Lactobacillus rhamnosus* IMC 501^®^, *Lactobacillus paracasei* IMC 502^®^ and SYNBIO^®^ against pathogens. J. Appl. Microbiol..

[56] Lucas J.A., Ramos Solano B., Montes F., Ojeda J., Megias M., Gutierrez Mañero F.J. (2009). Use of two PGPR strains in the integrated management of blast disease in rice (*Oryza sativa*) in Southern Spain. Field Crops Res..

[57] Battini F., Bernardi R., Turrini A., Agnolucci M., Giovannetti M. (2016). *Rhizophagus intraradices* or its associated bacteria affect gene expression of key enzymes involved in the rosmarinic acid biosynthetic pathway of basil. Mycorrhiza.

[58] Penrose D.M., Glick B. (2003). Methods for isolating and characterizing ACC deaminase-containing plant growth-promoting rhizobacteria. Physiol. Plant..

[59] Gilbert J.A., Hill P.J., Dodd C.E.R., Laybourn-Parry J. (2004). Demonstration of antifreeze protein activity in Antarctic lake bacteria. Microbiology.

[60] Rademaker J.L.W., Norman D.J., Forster R.L., Louws F.J., Schultz M.H., De Bruijn F.J. (2006). Classification and identification of *Xanthomonas translucens* isolates, including those pathogenic to ornamental asparagus. Phytopathology.

[61] Adhikari T.B., Gurung S., Hansen J.M., Bonman J.M. (2012). Pathogenic and genetic diversity of *Xanthomonas translucens* pv. *undulosa* in North Dakota. Phytopathology.

[62] Bentley S.D., Corton C., Brown S.E., Barron A., Clark L., Doggett J., Harris B., Ormond D., Quail M.A., May G. (2008). Genome of the actinomycete plant pathogen *Clavibacter michiganensis* subsp. *sepedonicus* suggests recent niche adaptation. J. Bacteriol..

[63] Méndez V., Valenzuela M., Salvà-Serra F., Jaén-Luchoro D., Besoain X., Moore E.R.B., Seeger M. (2020). Comparative genomics of pathogenic *Clavibacter michiganensis* subsp. *michiganensis* strains from Chile reveals potential virulence features for tomato plants. Microorganisms.

[64] Knief C., Delmotte N., Chaffron S., Stark M., Innerebner G., Wassmann R., Von Mering C., Vorholt J.A. (2012). Metaproteogenomic analysis of microbial communities in the phyllosphere and rhizosphere of rice. ISME J..

[65] Dong C.J., Wang L.L., Li Q., Shang Q.M. (2019). Bacterial communities in the rhizosphere, phyllosphere and endosphere of tomato plants. PLoS ONE.

[66] Zhou Q., Zhang X., He R., Wang S., Jiao C., Huang R., He X., Zeng J., Zhao D. (2019). The composition and assembly of bacterial communities across the rhizosphere and phyllosphere compartments of phragmites Australis. Diversity.

[67] Idris R., Trifonova R., Puschenreiter M., Wenzel W.W., Sessitsch A. (2004). Bacterial communities associated with flowering plants of the Ni hyperaccumulator *Thlaspi goesingense*. Appl. Environ. Microbiol..

[68] Castillo U.F., Browne L., Strobel G., Hess W.M., Ezra S., Pacheco G., Ezra D. (2007). Biologically active endophytic streptomycetes from *Nothofagus* spp. and other plants in Patagonia. Microb. Ecol..

[69] Navarro-Noya Y.E., Hernández-Mendoza E., Morales-Jiménez J., Jan-Roblero J., Martínez-Romero E., Hernández-Rodríguez C. (2012). Isolation and characterization of nitrogen fixing heterotrophic bacteria from the rhizosphere of pioneer plants growing on mine tailings. Appl. Soil Ecol..

[70] Tani A., Sahin N., Kimbara K. (2012). *Methylobacterium gnaphalii* sp. nov., isolated from leaves of *Gnaphalium spicatum*. Int. J. Syst. Evol. Microbiol..

[71] Ávila Martínez E.G., Lizarazo Forero L.M., Cortés Pérez F. (2015). Promoción del crecimiento de *Baccharis macrantha* (Asteraceae) con bacterias solubilizadoras de fosfatos asociadas a su rizósfera. Acta Biol. Colomb..

[72] Sánchez-López A.S., Ma Del Carmen A.G.C., Solís-Domínguez F.A., Carrillo-González R., Rosas-Saito G.H. (2018). Leaf epiphytic bacteria of plants colonizing mine residues: Possible exploitation for remediation of air pollutants. Front. Microbiol..

[73] Zhang Q., Acuña J.J., Inostroza N.G., Mora M.L., Radic S., Sadowsky M.J., Jorquera M.A. (2019). Endophytic bacterial communities associated with roots and leaves of plants growing in Chilean extreme environments. Sci. Rep..

[74] Roberts S.J., Preece T.F. (1984). A note on *Pseudomonas syringae* pv. *berberidis* infections of *Berberis*: Aetiology of a leaf spot and leaf fall disease in England. J. Appl. Microbiol..

[75] Russell G.B., Bowers W.S., Keesing V., Niemeyer H.M., Sevenet T., Vasanthaverni S., Wratten S.D. (2000). Patterns of bioactivity and herbivory on *Nothofagus* species from Chile and New Zealand. J. Chem. Ecol..

[76] Mølgaard P., Holler J.G., Asar B., Liberna I., Rosenbæk L.B., Jebjerg C.P., Jørgensen L., Lauritzen J., Guzman A., Adsersen A. (2011). Antimicrobial evaluation of Huilliche plant medicine used to treat wounds. J. Ethnopharmacol..

[77] González B., Vogel H., Razmilic I., San Martín J., Doll U. (2012). Biomass, resin and essential oil content and their variability in natural populations of the Chilean crude drug “bailahuén” (*Haplopappus* spp.). Bol. Latinoam. Caribe Plantas Med. Aromát..

[78] Ranković B., Kosanić M., Stanojković T., Vasiljević P., Manojlović N. (2012). Biological activities of *Toninia candida* and *Usnea barbata* together with their norstictic acid and usnic acid constituents. Int. J. Mol. Sci..

[79] Urzúa A., Echeverría J., Espinoza J. (2012). Lipophilicity and antibacterial activity of flavonols: Antibacterial activity of resinous exudates of *Haplopappus litoralis*, *H. chrysantemifolius* and *H. scrobiculatus*. Bol. Latinoam. Caribe Plantas Med. Aromát..

[80] Nikolić M., Marković T., Mojović M., Pejin B., Savić A., Perić T., Marković D., Stević T., Soković M. (2013). Chemical composition and biological activity of *Gaultheria procumbens* L. essential oil. Ind. Crops Prod..

[81] Concha J., Cavieres L.A., Sotes G.J., Hernández V. (2014). Essential oil composition of *Baccharis linearis* (Ruiz & Pav.) Pers. and *Baccharis paniculata* DC. leaves from Chile. Am. J. Essent. Oils Nat. Prod..

[82] Pandey B.P., Thapa R., Upreti A. (2017). Chemical composition, antioxidant and antibacterial activities of essential oil and methanol extract of *Artemisia vulgaris* and *Gaultheria fragrantissima* collected from Nepal. Asian Pac. J. Trop. Med..

[83] Chaudhary D.K., Khulan A., Jaisoo K. (2019). Development of a novel cultivation technique for uncultured soil bacteria. Sci. Rep..

[84] Acuña J.J., Marileo L.G., Araya M.A., Rilling J.I., Larama G.A., Mora M.L., Epstein S., Jorquera M.A. (2020). In situ cultivation approach to increase the culturable bacterial diversity in the rhizobiome of plants. J. Soil Sci. Plant Nutr..

[85] Roh S.W., Quan Z.X., Do Nam Y., Chang H.W., Kim K.H., Kim M.K., Im W.T., Jin L., Kim S.H., Lee S.T. (2008). *Pedobacter agri* sp. nov., from soil. Int. J. Syst. Evol. Microbiol..

[86] Ryan R.P., Monchy S., Cardinale M., Taghavi S., Crossman L., Avison M.B., Berg G., van der Lelie D., Dow J.M. (2009). The versatility and adaptation of bacteria from the genus *Stenotrophomonas*. Nat. Rev. Microbiol..

[87] Melnick R.L., Suárez C., Bailey B.A., Backman P.A. (2011). Isolation of endophytic endospore-forming bacteria from *Theobroma cacao* as potential biological control agents of cacao diseases. Biol. Control.

[88] Silby M.W., Winstanley C., Godfrey S.A.C., Levy S.B., Jackson R.W. (2011). *Pseudomonas* genomes: Diverse and adaptable. FEMS Microbiol. Rev..

[89] Petersen L.M., Tisa L.S. (2013). Friend or foe? a review of the mechanisms that drive *Serratia* towards diverse lifestyles. Can. J. Microbiol..

[90] Liu Q., Liu H.C., Zhou Y.G., Xin Y.H. (2019). Microevolution and adaptive strategy of psychrophilic species *Flavobacterium bomense* sp. nov. isolated from glaciers. Front. Microbiol..

[91] Busse H.J. (2016). Review of the taxonomy of the genus *Arthrobacter*, emendation of the genus *Arthrobacter sensu lato*, proposal to reclassify selected species of the genus *Arthrobacter* in the novel genera *Glutamicibacter* gen. nov., *Paeniglutamicibacter* gen. nov., *Pseudoglutamicibacter gen*. nov., *Paenarthrobacter gen.* nov., and *Pseudarthrobacter gen*. nov., and emended description of *Arthrobacter roseus*. Int. J. Syst. Evol. Microbiol..

[92] Cid F.P., Maruyama F., Murase K., Graether S.P., Larama G., Bravo L.A., Jorquera M.A. (2018). Draft genome sequences of bacteria isolated from the *Deschampsia antarctica* phyllosphere. Extremophiles.

[93] Oliver A., Kay M., Cooper K.K. (2018). Comparative genomics of cocci-shaped *Sporosarcina* strains with diverse spatial isolation. BMC Genom..

[94] Abraham W.R., Strömpl C., Meyer H., Lindholst S., Moore E.R., Christ R., Vancanneyt M., Tindall B.J., Bennasar A., Smit J. (1999). Phylogeny and polyphasic taxonomy of *Caulobacter* species. Proposal of *Maricaulis* gen. nov. with *Maricaulis maris* (Poindexter) comb. nov. as the type species, and emended description of the genera *Brevundimonas* and *Caulobacter*. Int. J. Syst. Bacteriol..

[95] Tak E.J., Kim P.S., Hyun D.W., Kim H.S., Lee J.Y., Kang W., Sung H., Shin N.R., Kim M.S., Whon T.W. (2018). Phenotypic and genomic properties of *Brachybacterium vulturis* sp. nov. and *Brachybacterium avium* sp. nov. Front. Microbiol..

[96] Greene A.C., Euzéby J.P., Tindall B.J., Patel B.K.C. (2009). Proposal of *Frondihabitans* gen. nov. to replace the illegitimate genus name *Frondicola* Zhang et al. 2007. Int. J. Syst. Evol. Microbiol..

[97] Lee S.D. (2010). *Frondihabitans peucedani* sp. nov., an actinobacterium isolated from rhizosphere soil, and emended description of the genus *Frondihabitans* Greene et al. 2009. Int. J. Syst. Evol. Microbiol..

[98] Cardinale M., Grube M., Berg G. (2011). *Frondihabitans cladoniiphilus* sp. nov., an actinobacterium of the family *Microbacteriaceae* isolated from lichen, and emended description of the genus *Frondihabitans*. Int. J. Syst. Evol. Microbiol..

[99] Kim S.J., Lim J.M., Ahn J.H., Weon H.Y., Hamada M., Suzuki K.I., Ahn T.Y., Kwon S.W. (2014). Description of *Galbitalea soli* gen. nov., sp. nov., and *Frondihabitans sucicola* sp. nov. Int. J. Syst. Evol. Microbiol..

[100] Han S.R., Yu S.C., Kang S., Park H., Oh T.J. (2016). Complete genome sequence of *Frondihabitans* sp. strain PAMC28766, a novel carotenoid-producing and radiation-resistant strain isolated from an Antarctic lichen. J. Biotechnol..

[101] Eichenlaub R., Gartemann K.-H. (2011). The *Clavibacter michiganensis* subspecies: Molecular investigation of Gram-positive bacterial plant pathogens. Annu. Rev. Phytopathol..

[102] Timilsina S., Potnis N., Newberry E.A., Liyanapathiranage P., Iruegas-Bocardo F., White F.F., Goss E.M., Jones J.B. (2020). *Xanthomonas* diversity, virulence and plant–pathogen interactions. Nat. Rev. Microbiol..

[103] Kuddus M., Ramteke P.W. (2008). A cold-active extracellular metalloprotease from *Curtobacterium luteum* (MTCC 7529): Enzyme production and characterization. J. Gen. Appl. Microbiol..

[104] Undabarrena A., Beltrametti F., Claverías F.P., González M., Moore E.R.B., Seeger M., Cámara B. (2016). Exploring the diversity and antimicrobial potential of marine actinobacteria from the Comau Fjord in Northern Patagonia, Chile. Front. Microbiol..

[105] Yadav A.N., Sachan S.G., Verma P., Saxena A.K. (2016). Bioprospecting of plant growth promoting psychrotrophic Bacilli from the cold desert of north western Indian Himalayas. Indian J. Exp. Biol..

[106] Yadav A.N., Yadav N., Sachan S.G., Saxena A.K. (2019). Biodiversity of psychrotrophic microbes and their biotechnological applications. J. Appl. Biol. Biotechnol..

[107] Ortíz-Ojeda P., Ogata-Gutiérrez K., Zúñiga-Dávila D. (2017). Evaluation of plant growth promoting activity and heavy metal tolerance of psychrotrophic bacteria associated with maca (*Lepidium meyenii* Walp.) rhizosphere. AIMS Microbiol..

[108] Lalucat J., Mulet M., Gomila M., García-Valdés E. (2020). Genomics in bacterial taxonomy: Impact on the genus *Pseudomonas*. Genes.

[109] Sitaraman R. (2015). *Pseudomonas* spp. as models for plant-microbe interactions. Front. Plant Sci..

[110] Hernández M., Villalobos P., Morgante V., González M., Reiff C., Moore E., Seeger M. (2008). Isolation and characterization of a novel simazine degrading bacterium from agricultural soil of Central Chile, *Pseudomonas* sp. MHP41. FEMS Microbiol. Lett..

[111] Canchignia H., Altimira F., Montes C., Sánchez E., Tapia E., Miccono M., Espinoza D., Aguirre C., Seeger M., Prieto H. (2017). Candidate nematicidal proteins in a new *Pseudomonas veronii* isolate identified by its antagonistic properties against *Xiphinema index*. J. Gen. Appl. Microbiol..

[112] Hernández M., Morgante V., Ávila M., Villalobos P., Millares P., González M., Seeger M. (2008). Novel s-triazine-degrading bacteria isolated from agricultural soils of Central Chile for herbicide bioremediation. Electron. J. Biotechnol..

[113] Hayward A.C., Fegan N., Fegan M., Stirling G.R. (2010). *Stenotrophomonas* and *Lysobacter*: Ubiquitous plant-associated gamma-proteobacteria of developing significance in applied microbiology. J. Appl. Microbiol..

[114] Altimira F., Yáñez C., Bravo G., González M., Rojas L.A., Seeger M. (2012). Characterization of copper-resistant bacteria and bacterial communities from copper-polluted agricultural soils of Central Chile. BMC Microbiol..

[115] Wolf A., Fritze A., Hagemann M., Berg G. (2002). *Stenotrophomonas rhizophila* sp. nov., a novel plant-associated bacterium with antifungal properties. Int. J. Syst. Evol. Microbiol..

[116] Lindow S.E. (1983). Methods of preventing frost injury through control of epiphytic ice nucleation active bacteria. Plant Dis..

[117] Subramoni S., Nathoo N., Klimov E., Yuan Z.C. (2014). *Agrobacterium tumefaciens* responses to plant-derived signaling molecules. Front. Plant Sci..

[118] Wang C.J., Yang W., Wang C., Gu C., Niu D.D., Liu H.X., Wang Y.P., Guo J.H. (2012). Induction of drought tolerance in cucumber plants by a consortium of three plant growth-promoting rhizobacterium strains. PLoS ONE.

[119] Molina-Romero D., Baez A., Quintero-Hernández V., Castañeda-Lucio M., Fuentes-Ramírez L.E., Bustillos-Cristales M.D.R., Rodríguez-Andrade O., Morales-García Y.E., Munive A., Muñoz-Rojas J. (2017). Compatible bacterial mixture, tolerant to desiccation, improves maize plant growth. PLoS ONE.

[120] Saikia J., Sarma R.K., Dhandia R., Yadav A., Bharali R., Gupta V.K., Saikia R. (2018). Alleviation of drought stress in pulse crops with ACC deaminase producing rhizobacteria isolated from acidic soil of Northeast India. Sci. Rep..

[121] Gupta S., Pandey S. (2019). ACC deaminase producing bacteria with multifarious plant growth promoting traits alleviates salinity stress in French Bean (*Phaseolus vulgaris*) plants. Front. Microbiol..

[122] Olanrewaju O.S., Babalola O.O. (2019). Bacterial consortium for improved maize (*Zea mays* L.) production. Microorganisms.

[123] Kawahara H., Nakano Y., Omiya K., Muryoi N., Nishikawa J., Obata H. (2004). Production of two types of ice crystal-controlling proteins in Antarctic bacterium. J. Biosci. Bioeng..

[124] Yu S.O., Brown A., Middleton A.J., Tomczak M.M., Walker V.K., Davies P.L. (2010). Ice restructuring inhibition activities in antifreeze proteins with distinct differences in thermal hysteresis. Cryobiology.

[125] Nadeem S.M., Naveed M., Ayyub M., Khan M.Y., Ahmad M., Zahir Z.A. (2016). Potential, limitations and future prospects of *Pseudomonas* spp. for sustainable agriculture and environment: A review. Soil Environ..

[126] Subramanian P., Mageswari A., Kim K., Lee Y., Sa T. (2015). Psychrotolerant endophytic *Pseudomonas* sp. strains OB155 and OS261 induced chilling resistance in tomato plants (*Solanum lycopersicum* Mill.) by activation of their antioxidant capacity. Mol. Plant-Microbe Interact..

[127] Chase A.B., Arevalo P., Polz M.F., Berlemont R., Martiny J.B.H. (2016). Evidence for ecological flexibility in the cosmopolitan genus *Curtobacterium*. Front. Microbiol..

[128] Díez-Méndez A., Rivas R. (2017). Improvement of saffron production using *Curtobacterium herbarum* as a bioinoculant under greenhouse conditions. AIMS Microbiol..

[129] Mayer E., de Quadros P.D., Fulthorpe R. (2019). *Plantibacter flavus*, *Curtobacterium herbarum*, *Paenibacillus taichungensis*, and *Rhizobium selenitireducens* endophytes provide host-specific growth promotion of *Arabidopsis thaliana*, basil, lettuce, and bok choy plants. Appl. Environ. Microbiol..

[130] Barriuso J., Ramos Solano B., Gutiérrez Mañero F.J. (2008). Protection against pathogen and salt stress by four plant growth-promoting rhizobacteria isolated from *Pinus* sp. on *Arabidopsis thaliana*. Phytopathology.

[131] Cardinale M., Ratering S., Suarez C., Zapata Montoya A.M., Geissler-Plaum R., Schnell S. (2015). Paradox of plant growth promotion potential of rhizobacteria and their actual promotion effect on growth of barley (*Hordeum vulgare* L.) under salt stress. Microbiol. Res..

[132] Khan M.A., Asaf S., Khan A.L., Ullah I., Ali S., Kang S.M., Lee I.J. (2019). Alleviation of salt stress response in soybean plants with the endophytic bacterial isolate *Curtobacterium* sp. SAK1. Ann. Microbiol..

[133] Vimal S.R., Patel V.K., Singh J.S. (2019). Plant growth promoting *Curtobacterium albidum* strain SRV4: An agriculturally important microbe to alleviate salinity stress in paddy plants. Ecol. Indic..

[134] Irizarry I., White J.F. (2017). Application of bacteria from non-cultivated plants to promote growth, alter root architecture and alleviate salt stress of cotton. J. Appl. Microbiol..

[135] Berg M., Koskella B. (2018). Nutrient- and dose-dependent microbiome-mediated protection against a plant pathogen. Curr. Biol..

[136] Ma J., Zhang Q., Chen F., Zhu Q., Wang Y., Liu G. (2020). Remediation of resins-contaminated soil by the combination of electrokinetic and bioremediation processes. Environ. Pollut..

[137] Janisiewicz W.J., Buyer J.S. (2010). Culturable bacterial microflora associated with nectarine fruit and their potential for control of brown rot. Can. J. Microbiol..

[138] Martins G., Lauga B., Miot-Sertier C., Mercier A., Lonvaud A., Soulas M.L., Soulas G., Masneuf-Pomarède I. (2013). Characterization of epiphytic bacterial communities from grapes, leaves, bark and soil of grapevine plants grown, and their relations. PLoS ONE.

[139] Kandel S.L., Firrincieli A., Joubert P.M., Okubara P.A., Leston N.D., McGeorge K.M., Mugnozza G.S., Harfouche A., Kim S.H., Doty S.L. (2017). An in vitro study of bio-control and plant growth promotion potential of *Salicaceae* endophytes. Front. Microbiol..

[140] Zhou X., Wang J.T., Zhang Z.F., Li W., Chen W., Cai L. (2020). Microbiota in the rhizosphere and seed of rice from China, with reference to their transmission and biogeography. Front. Microbiol..

